# The Effects of Moderate and Severe Salinity on Composition and Physiology in the Biomass Crop *Miscanthus × giganteus*

**DOI:** 10.3390/plants9101266

**Published:** 2020-09-25

**Authors:** Evangelia Stavridou, Richard J. Webster, Paul R. H. Robson

**Affiliations:** 1 Institute of Biological, Environmental and Rural Sciences, Aberystwyth University, Aberystwyth SY23 3EE, UK; estavrid@certh.gr (E.S.); R.J.Webster1@ljmu.ac.uk (R.J.W.); 2 Institute of Applied Biosciences, Centre for Research and Technology-Hellas, GR570 01 Thessaloniki, Greece; 3 School of Biological and Environmental Sciences, Liverpool John Moores University, Liverpool L3 3AF, UK

**Keywords:** Miscanthus, bioenergy, salinity tolerance, ion composition, osmoregulation, photosynthesis, biomass, combustion properties

## Abstract

Saline land represents a growing resource that could be utilised for growing biomass crops, such as *Miscanthus × giganteus* (Greef et Deu.), for eliminating competition with staple food crops. However, the response mechanisms to different salinity regimes, in relation to the impact on quality of the harvested biomass and the combustion properties are largely unknown. Herein, the focus was on the salt-induced compositional changes of ion flux and compartmentalization in the rhizome, stems, and leaves in relation to their impact on salinity tolerance and the combustion quality through investigating the photophysiological, morphophysiological, and biochemical responses of *M. × giganteus* to moderate and a severe salinity. Severe salinity induced an immediate and sustained adverse response with a reduction in biomass yield, photoinhibition, and metabolic limitations in photosynthesis. Moderate salinity resulted in a slower cumulative response with low biomass losses. Biomass composition, variations in ion compartmentalisation and induction of proline were dependent on the severity and duration of salinity. Ash behaviour indices, including the base percentage and base-to-acid ratio, indicated lower corrosion potential and lower risk of slagging under salinity. Understanding the impact of salinity on the potential for growth on saline land may identify new targets for breeding salinity-tolerant bioenergy crops.

## 1. Introduction

Degraded lands, often termed as marginal, have been reported suitable for cultivation of grasses, which are more adapted to low-nutrient, erodible, or drought prone soils [1]. Second generation perennial biomass crops, such as the grass Miscanthus, which is a highly productive and sustainable crop for bioenergy and feedstock for the bioeconomy [2], are ideal for cultivation on marginal land and would not compete with conventional food crops [3]. Saline land is marginal for most agriculture and represents a growing resource that could be utilised for Miscanthus cultivation [4]. *Miscanthus sinensis* exhibits salt spray tolerance growing in coastal landscapes as ornamental grass [5,6], with salt concentrations higher than 10 dS m^−1^ NaCl reducing the yield by over 50% [7]. The genetic diversity of salt tolerance to combinations of salinity and drought conditions [8] and single salt stress in *Miscanthus* have been recently documented [9,10,11]. Nevertheless, studies of salt tolerance mechanisms in *Miscanthus* have focused on the morphophysiological and biochemical [7,8,10,11,12,13] and transcriptional [14] responses.

Plant growth under salinity stress is mainly affected by an initial lower water potential associated with concentrated solutes in the root zone—an osmotic stress similar to water deficit, followed by an ionic imbalance, which occurs as salts perturb the uptake of beneficial nutrients, and under prolonged salt stress the excessive ion accumulation may lead to toxicity [15,16,17]. Salt stress affects plant metabolism or susceptibility to injury through complex interacting processes. Nutrient imbalance is induced due to hyper-ionic and hyperosmotic stress during ion uptake and circulation of ions within the plant and the accumulation of Na^+^ and Cl^−^ ions at toxic levels inside the cytoplasm [18]. These conditions result in nutrient imbalance, membrane injury, altered levels of phytohormones/osmoprotectants, inhibition of enzyme activity regulation, and disfunction of metabolic processes, including photosynthesis, by disrupting cellular homeostasis, and could ultimately lead to plant death [19,20]. However, sodium is essential for plant metabolism, osmotic potential, and turgor maintenance, and has been used to increase yield in several semi-halophytic (sugar-beets) and glycophytic (tomato) [21] crops and is considered important for several C4 species [22,23], but this essential or even beneficial role of sodium, has not yet been investigated in NADP-ME type C4 species, such as *Zea mays*, *Sorghum bicolor* [24], *M. × giganteus*, and *Saccharum officinarum* [25]. Similarly, chloride (Cl^−^) is involved in the regulation of enzymatic activities, such as the Cl^−^-dependent α-amylases and angiotensin-converting enzyme (ACE) [26], maintenance of turgor and pH, and is also a co-factor in photosynthesis [20,27]. However, at high concentrations, chloride has toxic effects to plants and impairs chlorophyll production, which induces chlorotic symptoms [20].

Different characteristics, which are species and developmental stage dependent, may contribute to salinity tolerance [16,28,29]. An effective strategy for glycophytes to cope with salt stress is to keep low cytosolic and shoot Na^+^ concentrations at the cellular and whole plant level respectively, by controlling the ion influx into root xylem through the casparian strip [30]. By contrast, halophytes have developed barrier mechanisms reducing Na^+^ influx in roots [31,32] and tend to accumulate more Na^+^ in the shoot rather than the root [33]. The salt overly sensitive (SOS) pathway regulates Na^+^/K^+^ ion homeostasis under excess salt levels and maintains low cytoplasmic concentrations of sodium by sequestering Na^+^ in the vacuoles [34]. Halophytes tolerate high concentrations of intracellular ions having enhanced antioxidant capacity and resistant to salinity metabolic activity [35,36,37,38]. The level at which ROS causes oxidative damage varies between glycophytes and halophytes [39]. In halophytic species, non-enzymatic control of ROS homeostasis is achieved through the production and hyperaccumulation of antioxidants, such as ascorbate and glutathione [38], glycine-betaine (>90 μmol dry weight) [36], and proline [40,41]. In non-halophytes, salt tolerance involves the accumulation of compatible solutes like proline, soluble sugars, polyamines, and glycine-betaine in the cytosol and organelles to induce osmotic adjustment and osmoprotection [42,43]. Proline biosynthesis is considered an important mechanism of adaptation to mitigate the imbalance between light energy absorbed through photochemistry and energy used in electron transport chain and carbon metabolism under stress [44]. The photosynthetic performance of plants is usually suppressed by salinity [45,46] due to stomatal limitations or non-stomatal effects, which include low chlorophyll content and leaf senescence related to ion toxicity [47,48] along with alterations in leaf photochemistry and carbon metabolism [49]. High concentrations of NaCl do not affect the photochemical efficiency of PSII (*F*_v_/*F*_m_), indicating that possibly the primary photochemistry of PSII is unaffected under salinity [50]. Duration and intensity of salt stress mainly define the relative size of the impact of stomatal and non-stomatal limitations on photosynthetic performance [51,52].

For combustion, biomass should be low in moisture and inorganic elements. Several minerals (potassium (K), chlorine (Cl), nitrogen (N), calcium (Ca), sodium (Na), aluminium (Al), iron (Fe), silicon (Si), and sulphur (S)) have been shown to contribute in ash formation having a major effect on biomass thermal conversion efficiency [53,54]. Low ash melting behaviour can lead to slagging due to ash depositions in the boiler and fouling because of the heat transfer section [55]. The elemental composition varies significantly depending on the genotype, harvest time, geographical location, and the inorganic fertiliser application [56]. Harvest time is probably one of the most relevant factors, considering that delayed senescence and leaf fall reduces the content of ash producing leaves in the harvest [57] and that high ash content negatively impacts the yield and quality of fast pyrolysis liquids [58].

The response mechanisms of the commercial hybrid *M. × giganteus*, in relation to the impact of salinity on the quality of the harvested biomass and the combustion properties under moderate and high salinity are largely unknown. This study aims to determine the photophysiological and morphophysiological response of *M. × giganteus* to moderate and a severe salinity stress, coupled with the salt induced compositional changes in terms of ion influx and compartmentalization in the different plant tissues in relation to their impact on salinity tolerance and the combustion properties.

## 2. Results

### 2.1. Effects of Moderate and Severe Salinity on Plant Growth

**Figure 1:**
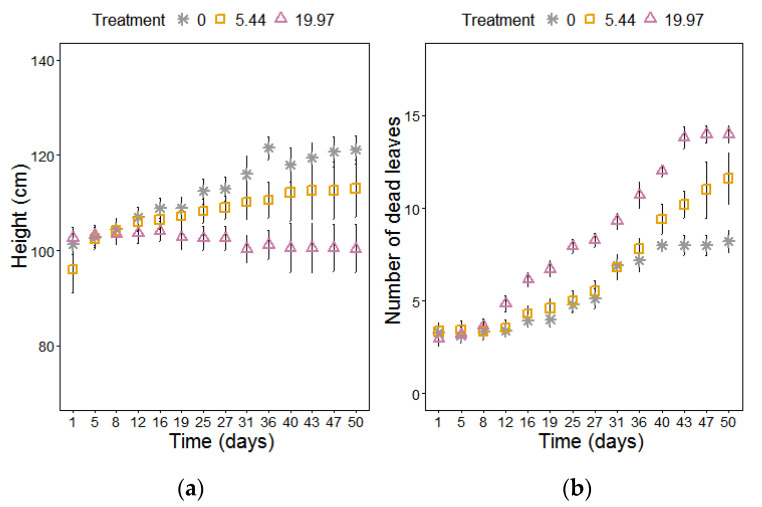
(**a**) Stem height and (**b**) number of senesced (dead) leaves of *M. × giganteus* at 0, 5.44, and 19.97 dS m^−1^ over a period of 54 days. Data are mean ± Standard Error (days 1–16: *n* = 20; days 17–26: *n* = 15; days 27–37: *n* = 10; days 38–54: *n* = 5).

Increased salinity negatively affected plant growth (Figure 1; Table 1) and biomass production (Table 1). High salt stress (19.97 dS m^−1^) induced an immediate and sustained adverse response, whereas the moderate salt stress (5.44 dS m^−1^) resulted in a slower cumulative response compared to the control plants (Supplementary Materials Figure S1). Height of the main stem (cm) was reduced under both salt treatments in response to time (*p* < 0.001) (Figure 1), with an earlier response observed at 19.97 dS m^−1^ compared to the moderate stress and control treated plants. The interaction effect between treatment and time (*p* < 0.001) on total number of senesced leaves, showed an early significant increase at 19.97 dS m^−1^ (*p* < 0.05), compared to the delayed senescence observed at 5.44 dS m^−1^ (Figure 1). The leaf area was significantly affected by the interaction effect between treatment and time (*p* < 0.001) at 19.97 dS m^−1^ NaCl with a decrease being observed between week 3–5 (*p* < 0.05) (Figure S2).

### 2.2. Biomass Accumulation in Response to Salinity

**Figure 2:**
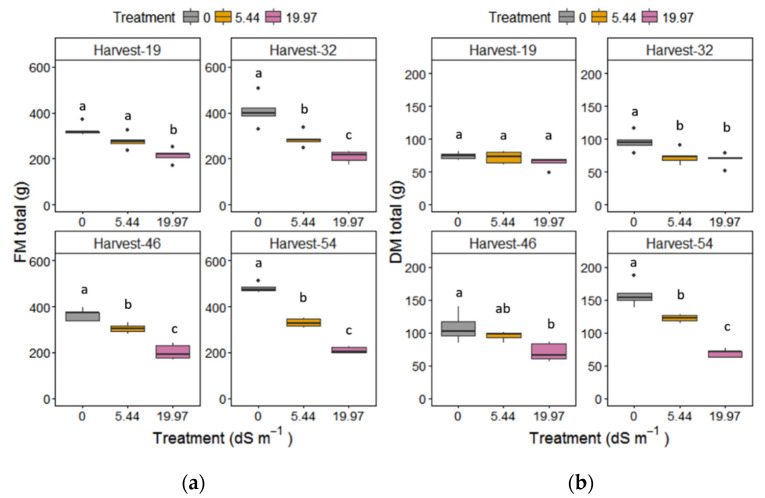
(**a**) FM total and (**b**) DM total (above and below biomass) of *M. × giganteus* at 0, 5.44, and 19.97 dS m^−1^ NaCl at the four harvest days (19, 32, 46, and 54). Data are mean ± Standard Error (*n* = 5). Different letters show significant differences between treatments within each day (*p* < 0.05). Dots indicate outliers (data points that are located outside the fences, “whiskers” of the boxplot).

Leaf number was significantly reduced under moderate and high NaCl stress across time (*p* < 0.05); however, the stem number was unaffected by salinity, harvest time, and their interaction (data not shown). The total production of fresh matter (FM) and dry matter (DM) was reduced in response to treatment (*p* < 0.001), especially at severe salinity (*p* < 0.05) after harvest day 32 (Figure 2; Table 1 and Table S1). Across time, only plants under moderate salinity and control conditions increased their total DM. Aboveground DM was significantly reduced under severe salinity, whereas no changes were observed under moderate salinity (Table 2). DM of leaves and stems was also reduced in plants treated with both salt treatments after harvest day 32 and showed an increase only in moderate salinity at the last harvest point (Table 2). Belowground DM was reduced significantly in severe salinity at harvest days 46 and 54, due to a reduction in rhizome DM observed after harvest day 32 and a delayed decrease in roots DM under severe salinity at the final harvest day (Table 2).

### 2.3. Physiological Response to Salinity

**Figure 3:**
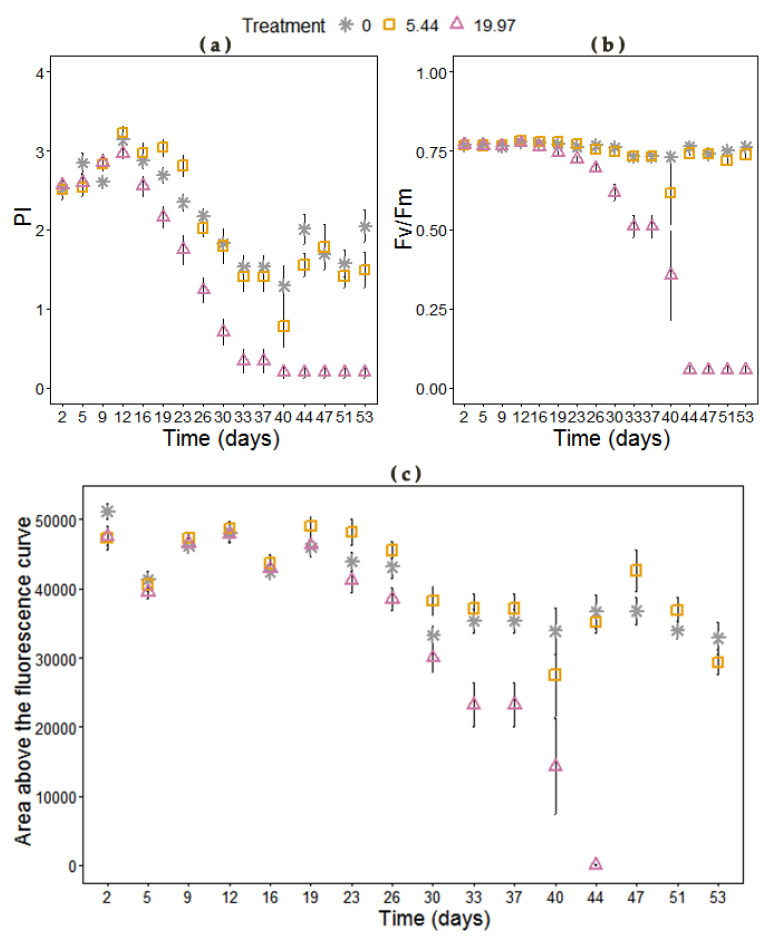
(**a**) Performance index, (**b**) maximum quantum yield of PSII (*F*_v_/*F*_m_) and (**c**) area above the fluorescence curve of *M. × giganteus* at 0, 5.44, and 19.97 dS m^−1^ over a period of 54 days. No values could be obtained from leaves after day 44 at the 19.97 dS m^−1^. Data are mean ± Standard Error (days 1–16: *n* = 20; days 17–26: *n* = 15; days 27–37: *n* = 10; days 38–54: *n* = 5).

**Figure 4:**
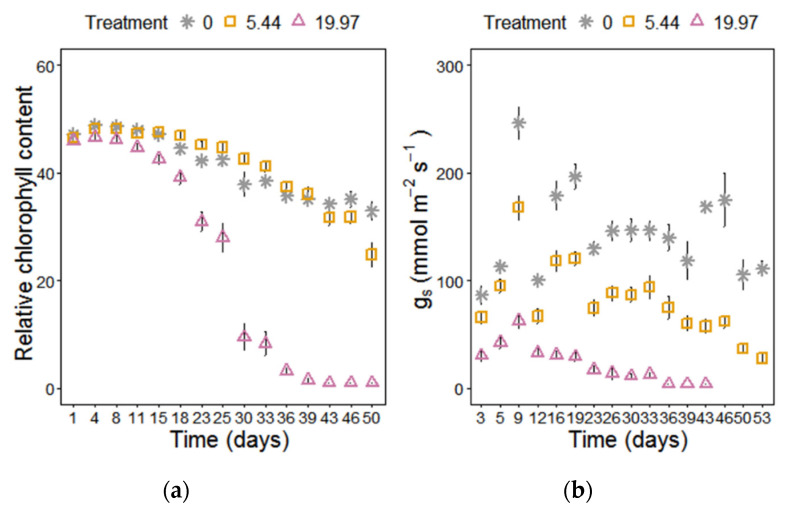
(**a**) Relative chlorophyll content and (**b**) stomatal conductance (*g*_s_) of *M. × giganteus* at 0, 5.44, and 19.97 dS m^−1^ over a period of 54 days. For the stomatal conductance measurements, no values could be obtained from leaves after day 43 at the 19.97 dS m^−1^. Data are mean ± Standard Error (days 1–16: *n* = 20; days 17–26: *n* = 15; days 27–37: *n* = 10; days 38–54: *n* = 5).

Several physiological parameters were affected by the cumulative effect of salinity (Table S2.). The significant effect of salinity on PSII maximum efficiency (*F*_v_/*F*_m_) was attributed not only to the effect of treatment per se but also on the duration of the treatment (effect of time) (Table S2). When taking into consideration these effects we observed that Miscanthus plants treated with moderate salinity were unaffected compared to controls and only severe salinity had a negative impact on PSII maximum efficiency after 23 days, with the impact becoming more severe with time, leading to complete inhibition of chlorophyll fluorescence (Figure 3). Performance index (PI) was a more sensitive indicator of photoinhibition for the highest salt concentration (19.97 dS m^−1^ NaCl) showing an earlier response, at 10-days, but this was unaffected by moderate salinity stress (Figure 3). The area above the fluorescence curve was significantly reduced after day 40 only in the highest salinity (Figure 3), indicating that electron flow into the plastoquinone (PQ) pool on the reducing side of PSII was blocked. Relative chlorophyll content was significantly reduced under the accumulative impact of salinity (Figure 4) and the plants under moderate salinity showed a delayed reduction of chlorophyll content as observed on day 50 compared to the control plants (Figure S1). Increasing salinity induced a significant and immediate decrease in stomatal conductance (*g*_s_) (*p* < 0.001) with differences between the treatments being observed on day 3 under severe salinity and a week later in the moderate salt stress compared to control plants (Figure 4).

### 2.4. Effect of Salinity on Carbon Fixation Efficiency

**Figure 5:**
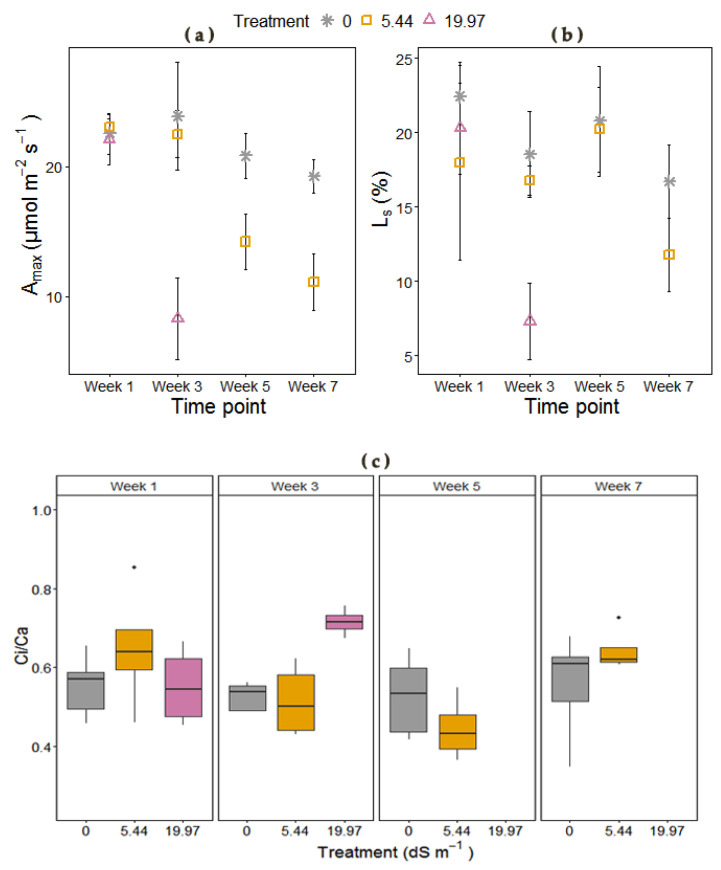
(**a**) Carbon assimilation rate (*A*_max_), (**b**) stomatal limitation (*L*s) both derived from the non-rectangular hyperbola model fitted to the A/Ci response of *M. × giganteus* at 0, 5.44, and 19.97 dS m^−1^ over four time points (Weeks: 1, 3, 5, and 7) and (**c**) ratio of intercellular to external CO_2_ concentration (C_i_/C_a_) at 0, 5.44, and 19.97 dS m^−1^ over four time points (Weeks: 1, 3, 5, and 7), measured at 390 μmol mol^−1^ CO_2_, 1500 μmol photon m^2^ s^−1^ and 26.4 °C in the controlled growth chamber environment. Dots indicate outliers (data points that are located outside the fences, “whiskers” of the boxplot). Data are mean ± Standard Error (0 dS m^−1^; *n* = 5, 5.44 dS m^−1^; *n* = 5 and 19.97 dS m^−1^; *n* = 4).

The effects of salt treatments on carbon fixation efficiency were investigated to describe the parameters derived from the dependence of CO_2_ assimilation rate (*A*) to leaf internal CO_2_ mole fraction (Ci). The measured for each plant at four time points and modeled *A*/C_i_ curves are presented in Figure S4. Severe salinity at harvest days 46 and 54 had a negative impact on plants, which were senesced and dry, and therefore the measurements would have been biased. The *A*_max_ (Figure 5 and Table S3) was significantly affected by the increasing salinity, with the highest salt concentration showing a more rapid effect on week 3, whereas moderate salinity-induced a delayed decline in *A*_max_ on week 5 (Table S3). The accumulative effect of salinity induced an increase in the ratio of intercellular to external CO_2_ concentration (C_i_/C_a_) at week 3, yet non-significant (time × treatment; *p* < 0.1) (Figure 5). The maximum carboxylation efficiency (CE) was significantly reduced on week 3 under severe salinity, and it was unaffected in moderate salinity in comparison to the controls (Table S3). The CO_2_ saturated PEP carboxylation rate (V_pmax_; μmol m^−2^ s^−1^) and the CO_2_ compensation point were reduced with increasing salinity yet not significantly, whereas the PEPC Michaelis–Menten constant for CO_2_ (Kp) and the curvature (omega, ω) of the A/C_i_ curves were not affected by the increasing salinity, but rather by time. Despite the reduction in stomatal conductance over time for both salt treatments (Figure 4b), stomata were not the main limiting factor of carbon fixation at 5.44 dS m^−1^ NaCl, until after week 5, where a slight decoupling was observed (Figure 5). For the severe salinity, rapid decline in the assimilation rate was mainly caused by metabolic limitations (Table S3, Figure 5).

### 2.5. Water Relations Responses

**Figure 6:**
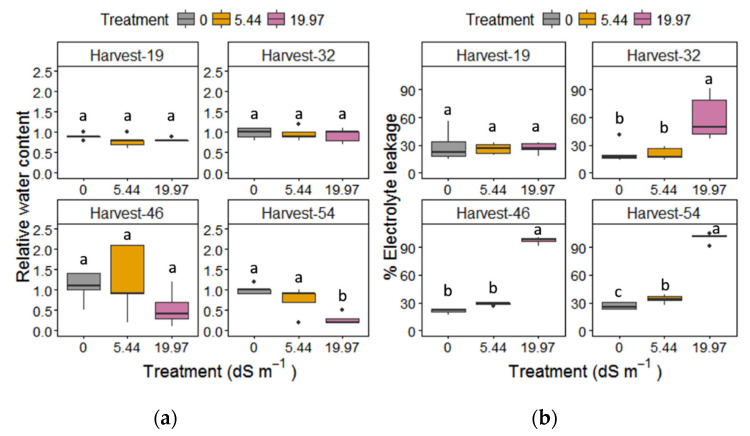
(**a**) Relative water content and (**b**) % electrolyte leakage of *M. × giganteus* leaves at 0, 5.44, and 19.97 dS m^−1^ NaCl on the four harvest days (19, 32, 46, and 54). Data are mean ± Standard Error (*n* = 5). Different letters show significant differences between treatments within each day (*p* < 0.05). Dots indicate outliers (data points that are located outside the fences, “whiskers” of the boxplot).

Intrinsic water use efficiency (WUEi) increased only in 5.44 dS m^−1^ NaCl-treated plants after week 3 (Table S3), followed by a significant decline at week 7. The relative water content (RWC) in the leaves of moderately stressed plants was not affected (Figure 6). However, under severe salinity, leaves showed a significant decrease in RWC at harvest day 54 (Figure 6).

### 2.6. Role of Salinity on Leaf Tissue Compounds

**Figure 7:**
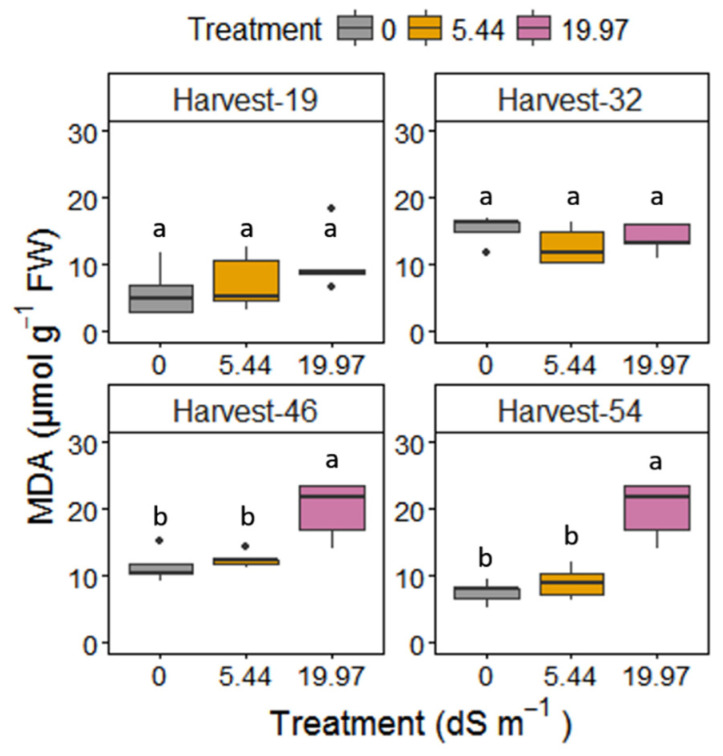
Malondialdehyde (MDA) content of *M. × giganteus* leaves at 0, 5.44, and 19.97 dS m^−1^ NaCl on the four harvest days (19, 32, 46, and 54). Data are mean ± Standard Error (*n* = 5). Different letters show significant differences between treatments within each day (*p* < 0.05). Dots indicate outliers (data points that are located outside the fences, “whiskers” of the boxplot).

Salinity affected all of the biochemical parameters measured with a significant interaction effect between treatment and harvest time (Table 1). Relative electrolyte leakage in leaves was significantly increased under both salt stresses; however, in moderate salt stress it occurred only at the last harvest day 54 (Figure 6). Malondialdehyde (MDA) content increased significantly in leaves (Figure 7) only under 19.97 dS m^−1^ NaCl on harvest days 46 and 54. Proline accumulation in leaves increased dramatically on day 32 under severe salinity (Table 3).

**Figure 8:**
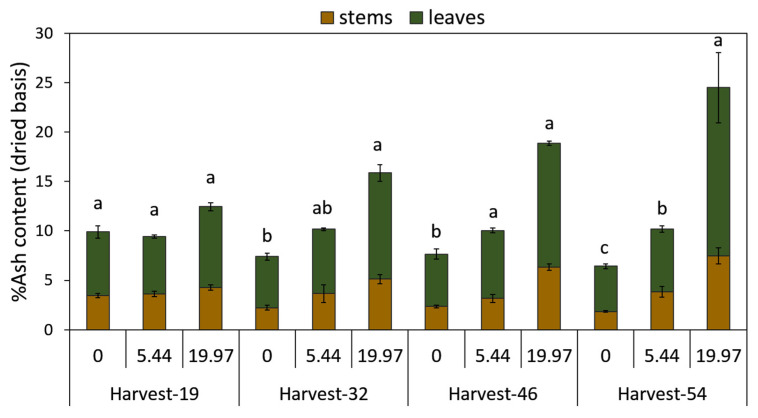
Ash content (%) of *M. × giganteus* leaves and stems at 0, 5.44, and 19.97 dS m^−1^ NaCl in the four harvest days (19, 32, 46, and 54) (*n* = 5). Different letters show significant differences between treatments within each day (*p* < 0.05). Data are mean ± Standard Error (Leaves: *n* = 3; Stems and Rhizomes: *n* = 5).

The ash content increased with salinity intensity in both, leaves and stems across harvest days (Figure 8; Table 4). The ash content was higher under severe salinity, relatively high under moderate salinity and low under control conditions. Between the two tissue types, leaves had a higher percentage in ash content compared to stems, regardless of the treatment and time effects (Figure 8; Table 4). Leaves in severe salinity, showed ash contents increased by 1.1-fold (harvest day 32) and 2.7-fold greater (harvest day 54), whilst the ash content of leaves from moderate salinity treatments increased by approximately 0.4-fold when compared to the controls on harvest day 54. The ash content in stems under severe salinity was 1.7-fold and 3-fold greater on harvest days 46 and 54, respectively, whereas under moderate salinity the increase was up to 1.1-fold, compared to control plants on harvest day 54 (Figure 8).

### 2.7. Ion Flux and the Role of Salinity on Tissue Compartementalisation and Combustion Properties

Salinity affected the levels of total K, Ca, Mg, S, Cl, and Si, but not Fe (Figure S4; Table 5, Tables S4 and S5). Sodium increased dramatically in leaves, stems, and rhizome under both salinity treatments with plants under severe salinity having a more dramatic effect on the Na accumulation. The distribution of Na was equal throughout the components of the biomass partitions in the control treatment. However, Na was concentrated in the rhizome in moderately stressed plants, and this was translocated and concentrated in the leaves in plants under severe salinity stress. High salinity stress produced higher total Na in treated plants during the experiment, but under moderate stress the increase was induced only after day 46 (Table S6). Water soluble chloride, unlike Na, increased only in leaves and rhizome under both salinity treatments throughout the experimental duration, but no effect was observed on the stems (Table 5 and Table S6). Accumulation of total potassium was also higher in leaves and stems, but only under severe salinity, yet Ca increased in leaves under both stress treatments and in stems of plants growing under severe salinity stress. Total magnesium increased only in the rhizome under the severe salinity. Total sulphur increased only in stems and rhizomes under severe salinity and was higher in rhizomes compared to the above-ground tissues. Silicon accumulated to higher levels in leaves and stems under 19.97 dS m^−1^ NaCl (Table 5). More Ca was translocated from the rhizome to the above-ground biomass, whereas total K reached the stems at moderate salinity, and the leaves under severe salinity (Table 5). Total potassium was significantly higher in leaves under severe salinity and did not show any change over time compared to the moderately stressed and the control plants, which had significantly lower K over time (Table S6). Total sulphur in leaves was reduced with increasing salinity, whereas only under moderate stress a reduction over harvesting points was observed. The Si and Ti were significantly affected only by the increasing intensity of salinity and the observed differences were detectable only at the final harvest day (day 54) (Table S6). The Si was mainly accumulated in the leaves with a lower content being found in the stems and no traces were detected in the rhizomes (Table 5).

The ratios K/Na and Ca/Na (Table 6 and Table S6) were reduced in leaves with increasing salinity in all tissue types and harvest days. Both ratios were higher in leaves compared to stems and rhizomes at moderate salinity; however, under severe salinity stress K/Na was higher in the rhizome compared to leaves and Ca/Na was higher in leaves compared to stems and rhizome. The early decrease observed in both ratios in leaves from harvest day 19 occurred under severe salinity, whilst at moderate salinity K/Na ratio showed a delayed decline (harvest day 54) compared to Ca/Na, which decreased eight days earlier (Harvest day 46). Lower Ca/K and Si/K ratios, and therefore increased slagging tendency, were only observed in leaves under severe salinity. Compared to the control and moderate salinity treatments, severe salinity induced a reduction in Ca/K, which began on harvest day 46 and was followed by a cumulative significant reduction in Si/K on the final harvest day 54 (Table 6 and Table S6).

The molar ratio 2S/Cl, which is used as empirical index to evaluate the corrosion potential for herbaceous biomass, was higher in stems compared to leaves (*p* < 0.001) on the final harvest day, whereas time course measurements in leaves showed that under both salt treatments the ratio 2S/Cl was significantly lower compared to the control pants. Significantly increased Rb/a is used as an indicator that the fouling tendency of a fuel ash increased under 19.97 dS m^−1^ NaCl in leaves (Table 7 and Table S7); however, in stems the highest salt level induced a decline in the Rb/a. However, stems compared to leaves, always showed higher base to acid ratio except under severe salinity, where no differences where observed (Table 7). The % base was greater in both leaves and stems with increasing salinity and especially under severe salinity stress. Leaves had the highest % base compared to stems, except in moderate salt stress, where no differences between the organs were observed (Table 7). The increase in % base of leaves occurred earlier (day 19) under severe salinity, whilst under moderate salinity the % base was also increased compared to control plants on day 54 (Table S7).

## 3. Discussion

Saline land provides an opportunity for growing second generation biomass crops to avoid competition with staple food crops; however, quantitative, and qualitative effects of salinity may be a constraint towards the utilisation of such lands for biomass yield. The potential to exploit salt affected lands will depend on the salt concentrations in the soil and the extent at which the yield and biomass quality are reduced. Understanding plant tolerance under salinity and its impacts on the harvested product may provide a new range of targets for breeding strategies towards salinity-tolerant bioenergy crops. Herein, we investigated how composition, including ion content and compartmentalization, proline accumulation, and water relations interact with photophysiological and morphophysiological response mechanisms towards salinity tolerance and biomass quality of *M. × giganteus*.

### 3.1. Effects of Moderate and Severe Salinity on Biomass Accumulation and Partitioning

Elevated salt content induced a reduction in the water uptake capacity of Miscanthus, observed as a rapid reduction in growth rate, in a similar way as in drought stress. Severe salinity (19.97 dS m^−1^), induced an immediate and sustained adverse response with a reduction in biomass yield up to 56.4%. Moderate salinity (5.44 dS m^−1^) triggered a slower cumulative response compared to the control plants but did not incur great losses in biomass (<23%). The reduction in above-ground DM was manifested as abscission of older leaves, highlighting a cumulative ionic effect due to prolonged exposure to salinity. High salinity induced early premature senescence of older leaves (from day 12), whilst the effect of moderate salinity was more gradual, suggesting plants responded initially to osmotic and later to accumulated ionic effect of salinity, compared to the severe ionic effect under high salinity. Consecutive harvests revealed root growth was inhibited earlier (day 19) under high NaCl, compared to the delayed inhibition observed in moderate salinity. Rhizome DM was also reduced earlier in plants growing under high salinity. Płażek et al. [13] observed a similar response in *M. × giganteus*. This ability of perennial grasses to maintain the below-ground biomass under stress conditions, could preserve sufficient reserves to invest in the following season’s growth [59]. This may be physiologically relevant for transitory stresses like drought; however, it remains unclear how the annual growth will be affected by maintaining the below-ground biomass under consistent salinity stress of variable intensities due to seasonal environmental changes [60,61].

### 3.2. Impact of Salinity on Metabolic and Non-Metabolic Factors

The degree of tolerance to the osmotic effects of salinity is reflected on the ability of plants to maintain the *g*_s_ [62], which is associated with regulation of CO_2_ assimilation rate and transpiration and is positively associated to the relative growth rate in saline soils [63,64]. Both salinity treatments induced a reduction in *g*_s_, which was more severe under 19.97 dS m^−1^ NaCl. Reduction of *g*_s_ has been attributed to the impact of high ion concentrations in leaves, the induced perturbation of water status and the local synthesis of abscisic acid in the guard cells [65]. In maize, the decline in the assimilation rate under increasing salinity was mainly associated to stomatal limitations (*L*s) and to a lesser extent photoinhibition [66]. Herein, high salinity induced a significant decrease in the photochemical efficiency of PSII (*F*_v_/*F*_m_). The elevated C_i_/C_a_ values (week 3), combined with reductions in V_pmax_, indicate that the decrease in carbon assimilation rate could not be explained by CO_2_ deficiency or limitations in stomatal function. The impact of salinity on photosynthesis is therefore likely due to the observed reductions in the activity and regeneration of PEP carboxylase, which was reflected in significant reduction in carbon assimilation rate and as a result significant losses in DM. Similar response patterns in physiological parameters have been previously observed in Miscanthus, switchgrass, and sugarcane in response to cold stress [67,68,69]. In moderate salinity the observed gradual reduction in carbon fixation (*A*_max_) occurred mainly due to salt-induced osmotic effect, manifested as induced stomatal resistance. The cumulative effect under prolonged exposure to moderate salinity resulted in uptake and accumulation of Na^+^, which according to Muranaka et al., [70] may have directly affected the electron transport; thus, the observed delayed reduction in photosynthetic capacity. However, this delayed reduction was not severe enough to affect biomass accumulation and increase in proline content may have played a significant role in that.

Severe salinity caused reduction in photosynthesis not only in terms of metabolic limitations but also photoinhibition. The decline in maximum quantum yield of PSII (*F*_v_/*F*_m_) was observed under high salinity treatment after day 23 with the impact becoming more severe over time, leading to complete inhibition of chlorophyll fluorescence. The reduction in the area above the fluorescence curve between *F*_o_ and *F*_m_ indicated that the electron flow to the plastoquinone (PQ) pool on the reducing side of PSII was blocked by high salinity as was demonstrated by Kalaji et al. [71] in barley, where 120 mM NaCl resulted in inhibition of electron transport from the reaction centres to the plastoquinone pool. In contrast, moderate salinity did not affect the maximum quantum yield of PSII, which could be the explained by the non-toxic accumulation of Cl in the leaves that is shown to activate PSII [26].

### 3.3. Proline Accumulation in Relation to Chlorophyll Content, Electrolyte Leakage, and Photosynthetic Performance

The foliar water relations are influenced by the ion accumulation and plant ability for osmotic adjustment, [72]. Herein, *M. × giganteus* was able to maintain leaf water and relative chlorophyll contents after prolonged moderate salinity stress, indicating a potential mechanism of osmotic adjustment, which is related to the accumulation of osmoprotectant molecules, such as proline. Proline is a multifunctional amino acid that adjusts the osmotic potential inside the cytoplasm and its accumulation during stress conditions is mainly due to increased synthesis and reduced degradation [73]. The increased proline accumulation in *M. × giganteus* at 19.97 dS m^−1^ NaCl, as early as the first harvest day (day 19), provides evidence for water preservation in leaves through osmotic adjustment. Although in high salinity, the osmotic adjustment occurs at the expense of plant growth, it may assist with plant survival or even recovery (reviewed by [65,74,75]). The increased proline accumulation observed under moderate salt stress appears to have conferred tolerance to the photosynthetic apparatus. The PSII maximum efficiency and the electron flow to the PQ pool on the reducing side of PSII, were unaffected by moderate salinity throughout the experiment and CO_2_ assimilation was unaffected up to week 3. However, in the high salt treatment, the excessive accumulation of proline occurred too slowly to prevent a reduction of the negative effects on photosynthetic performance (Figure 4, Figure 5, Figure 6 and Figure 7). Proline is synthesized under stress conditions both in the shoot and root and can also be transported to the root via the phloem by proline transporters [76]. Therefore, in this study, proline accumulation under salinity might contribute to root growth, which was maintained, and was only reduced at the highest salinity on the final harvest day (54). Proline homeostasis, rather than the proline accumulation per se, is considered important for the maintenance of cell division under abiotic stress [77]; however, the effect of the temporal and spatial concentrations of proline (basal versus elevated levels) on plant growth in response to stress is yet to be determined [77].

The pronounced leaf senescence observed under high salinity after day 15 can be initially induced by the osmotic phase of salinity, when growth inhibition and metabolic changes occur [15,28]. Herein, the ability of *M. × giganteus* to maintain water content of leaves under both salinity levels and leaf chlorophyll after prolonged stress duration at moderate salinity stress, may indicate a potential mechanism of osmotic adjustment in moderate salinity, such as salt-induced accumulation of proline. Relative electrolyte leakage increased in leaves exposed to 19.97 dS m^−1^ NaCl from harvest day 32 onwards, whereas a delayed increase (final harvest day) was observed at 5.44 dS m^−1^ NaCl treated plants. The premature leaf senescence of *M. × giganteus* and the damage to membrane structure induced by high salinity concentrations may be a result of the excessive ion accumulation in shoots and leaves, especially Cl^−^ and Na^+^ in toxic levels, which could also explain the reduction in photosynthesis. Both salinity treatments affected the ionic balance of *M. × giganteus* leaves, stems, and rhizomes (Figure S4; Table 6 and Table 7). In many plant species high NaCl concentrations act antagonistically to the uptake of nutrients like K^+^, Ca^2+^, and Mg^2+^, by reducing their concentrations [78,79,80]. The significant accumulation of sodium (Na) in leaves was observed only at the highest salinity treatment, whereas stems and rhizome accumulated total Na under both salinity treatments and especially at the highest salinity level. Total Na content did not differ in different organs in control plants, whilst it was sequestered in rhizome under 5.44 dS m^−1^ NaCl and was translocated in greater quantities to leaves over time under 19.97 dS m^−1^ NaCl. The observed alterations in ion flux and ion distribution among plant tissues were accompanied by the induction of proline in the leaves, possibly as a measure of osmotic adjustment. However, the impact of severe salinity was too intense for proline to counterbalance the negative effects from increased electrolyte leakage and lipid peroxidation.

### 3.4. Ion Accumulation and Compartmentalisation Ability

Elemental composition effects cellular processes and stress tolerance, but in biomass crops this is of particular importance in affecting biomass composition. Changes in elemental composition may have beneficial or detrimental effects on biomass quality depending on which organs are affected and the direction of change. Herein, ion accumulation varied in different tissue types (leaves, stems, and rhizomes) and the ability to compartmentalise toxic ions to specific plant tissues, which is dependent on the severity of salinity was also demonstrated.

Under high salinity, total sodium (Na) was accumulated in leaves, stems, and rhizome, whereas under moderate stress was mainly found in rhizome and stems. Total Na content did not differ in different organs in control plants, whilst it was sequestered in the rhizome under 5.44 dS m^−1^ NaCl and was translocated in greater amounts to leaves over time under 19.97 dS m^−1^ NaCl. Water soluble chloride (Cl), unlike total Na, increased only in leaves and rhizome under both salinity treatments, but no treatment effect was observed in the stems, possibly due to compartmentation in leaf vacuoles. Under control conditions, *M. × giganteus* stems accumulated most chloride (16.2 mg g^−1^), and therefore may be characterised as a moderately tolerant crop to Cl^−^ toxicity.

Maintaining Ca accumulation and transport under salinity is important for enhanced tolerance [81], as it modulates intracellular Na^+^ homeostasis in plants (Munns, 2002). In both salinity treatments, Ca was translocated from the rhizome to the above-ground biomass and particularly leaves, whereas total K was elevated in stems at moderate salinity and in the leaves under severe salinity, where it was maintained at high levels in all harvest points. This increase in both Ca and K in leaves under salinity stress may demonstrate an exertion to maintain the osmoregulation and function of cell membranes [82]. Enhanced tolerance to salt stress has been observed in the presence of a more efficient selective uptake of K^+^ and cellular compartmentation and distribution of K^+^ and Na^+^ in the shoots of barley [83,84,85]. Total K was accumulated in greater amounts than total Na in stems and rhizomes in both salinity treatments (harvest day 54); however, in leaves, more Na was sequestered at the highest salinity level after harvest day 46, when it was at toxic levels causing rapid senescence, inhibiting gas exchange and causing extreme electrolyte leakage and an increase in lipid peroxidation. Similarly, Si accumulated at higher levels in leaves and stems ranged from 5.5% at control conditions to 6.7% and 8.5% at 5.44, and 19.97 dS m^−1^ NaCl, respectively. Non-stressed *M. × giganteus* has showed Si content between 0.55 and 2.42% grown on various locations in US [86]. Despite the negative impact of Si on thermo-conversion efficiency of biomass to bioenergy, there are several beneficial biological effects, including enhanced photosynthetic activity, increased resistance to pests and pathogens, reduced mineral toxicity, improved nutrient balance, and tolerance against drought, and frost stress [87,88]. Increased accumulation of Si in plants has been shown to enhance growth under drought by reducing transpiration [89] as well as under salinity, partially due to Si-induced decrease in transpiration, and the disruption of the Na concentration in roots and flag leaves of wheat [90] and rice [91]. According to this, it is possible that the observed 6.7% Si accumulated in leaves under moderate salinity played a role in the maintenance of growth and possibly in the lower accumulation of Na in these leaves.

### 3.5. Biomass Quality and Combustion Properties

The biomass of *M. × giganteus* has been shown to have good combustion characteristics [92] compared to other lignocellulosic crops [93]. The ash content, as expected, was greater in leaves compared to stems and increased up to 2.7-fold and 3-fold, respectively, at severe salinity on the final harvest day. In moderate salinity, ash content increased by 0.4-fold in leaves and 1-fold in stems (Figure 8). High ash content has been shown to significantly reduce the energy output of biomass combustion [51]. In Miscanthus, the leaves contain higher mineral content and twofold the ash content compared to stems or reproductive organs [57], which was also observed herein under all treatments. As such, premature leaf senescence and leaf loss occurring under increasing salinity may contribute to improved quality of the harvestable biomass and thus, compensate for the total yield loss. However, despite the lower biomass quality for combustion due to high ash content, it is expected that after winter period additional loss of mineral content will occur from either senescence, leaf drop or leaching, and thus, *M. × giganteus* could be a good candidate for growing under moderate salinity levels.

The ash melting behaviour is greatly affected by the elemental composition of ash (Na, K, P, Cl, Si, and Ca) [94]. Miscanthus is considered to have a low ash melting temperature [95,96,97] possibly related to the concurrent occurrence of increased Si, K, and Ca [98]. K concentration in the biomass fuel is required to be in low levels, because of slagging risk [92]. In this study, Si and Ca, contents were mainly observed in leaves, whilst K was present in both leaves and stems. Therefore, leaves may contribute to the increase of ash melting point and reduction of the slagging potential. Reductions in the ratios of Ca/K and Si/K, and therefore increasing slagging tendency, was only observed in leaves at severe salinity. Hence, the increased leaf loss in both salinity treatments possibly may contribute to enhanced biofuel quality according to the similar results observed under regular, non-stressed growing conditions by Monti et al. [57].

To reduce emissions and lower the corrosion risks in conventional boilers, especially when the fuel is high in Cl (>1–2 g kg^−1^) and K (>5 g kg^−1^) and low in S (<2 g kg^−1^), the maximum steam temperature has to be at 450 °C, [92]. In this study the Cl, K, and S concentrations were much lower in both leaves and stems, similar to results in *M. × giganteus* by Monti at al. [57] under control conditions. Minor corrosion is likely to occur if 2S/Cl molar ratio in the fuel are >4 [99]. Herein, the corrosion potential based on the molar ratio 2S/Cl, was higher in stems (1.34) compared to leaves (0.28), on the final harvest day and was not affected by salinity treatments, indicating lower corrosion potential. Therefore, despite leaves having higher accumulation of ion contents their presence may contribute to the increase of ash melting point and reduction of the slagging potential. The greater increase in the proxy estimations of base to acid ratio (R_b/a_) of leaves under severe salinity in relation to the decline in stems, indicate lower risk of slagging in stems at high salinity treatment. Among organs, stems showed consistently higher base to acid ratio except in high salinity stress, where no differences were observed. Nevertheless, the ratio was higher under all treatments in relation to the recommended values of <0.5 for low risk of slagging and >1 indicative of severe slagging problems [55].

We have addressed key knowledge gaps in unravelling Miscanthus response mechanisms to moderate and high salinity, which could be the basis for enhancing crop adaptation to climate change. The concluding remarks that can be drawn from this research are (i) *M. × giganteus* is identified as tolerant to moderate salinity stress due to osmotic adjustment, and therefore can be cultivated in moderate salinity affected lands as a more energetically suitable bioenergy crop that would balance the energetic input in terms of fertilization and cultivation requirements, without diminishing the combustion quality; (ii) the effects of salinity on C4 photosynthesis are mediated by both stomatal and metabolic limitations depending on the salt concentration; (iii) ion accumulation varied in the type of tissue and the ability to compartmentalise toxic and essential ions to specific tissues was demonstrated; (iv) proline accumulation in leaves was induced by increasing salinity, which previous studies have shown to have an osmoregulatory role in protecting metabolic related photosynthetic processes reducing lipid peroxidation under moderate salinity; and (v) the duration and intensity of increasing salinity inhibited the production of biomass, which was unaffected by moderate salinity. The results presented herein revealed the potential for growth of *M × giganteus* on saline areas and may contribute to the wider understanding of the mechanistic effects of moderate and severe salinity on morphophysiology, photophysiology, composition, and biomass quality of *M. × giganteus*. This approach is expected to provide insights into new targets for breeding salinity-tolerant bioenergy crops by dissecting the salt-induced osmotic stress and ion toxicity effects in order to highlight the potential for growth of the biomass on underutilized or abandoned land. Conventional harvest under field conditions are recommended to better understand the effect of salinity on biomass quality and combustion properties, because composition may differ due to additional loss of mineral content from senescence, leaf drop, or leaching.

## 4. Materials and Methods

### 4.1. Plant Material and Experimental Design

The experiment was conducted at IBERS, Aberystwyth University, Wales, UK in controlled glasshouse conditions (Venlo) with 16- and 8-h day/night photoperiod from supplemental lighting with approximately 500 μmol photons m^2^ s^−1^ of photosynthetically active radiation and 25 and 15 °C day/night cycle, respectively. *M. × giganteus* plants from approximately 20 g rhizome pieces were established and grown in 6.2 L pots containing John Innes No. 2 compost (Levington^®^, Evergreen Garden Care Ltd., Surrey, UK). A homogeneous population was selected and grown to seven fully expanded leaves. Two different NaCl concentrations (5.44 and 19.97 dS m^−1^ equivalent to 60 and 210 mM NaCl, respectively) and zero salt content (control), selected from our previous work [7], as indicative for the induction of different responses in *M. × giganteus*, were supplied via irrigation. To avoid osmotic shock, increasing rates of 5.44 dS m^−1^ NaCl were applied gradually per day until all treatments reached the target concentration (approximately on day 18; Figure S5). Plants were irrigated with ½ strength Hoagland’s solution [100] every 2 weeks with the electrical conductivity adjusted to the experimental salt concentrations. Moisture content and electrical conductivity was measured as an average of three measurements per pot using a WET sensor (WET; Delta-T Devices Ltd., Cambridge, UK) inserted at three roughly equidistant points around the surface of the pot, and readings were recorded by a hand-held moisture meter (HH2 moisture meter; Delta-T Devices Ltd., Cambridge, UK). A total of 60 plants (3 pots/m^2^) were treated for 54 days in a completely randomised design with 20 biological replicates per treatment and four harvest time points, at 19, 32, 46, and 54 days for destructive measurements, with *n* = 5 biological replicates per treatment (days 1–16: *n* = 20; days 17–26: *n* = 15; days 27–37: *n* = 10; and days 38–54: *n* = 5). All morphological and physiological measurements were performed twice every week between 09:00 and 14.00 h.

### 4.2. Morphological Measurements

The number of senesced (dead) leaves was assessed by counting the leaves that were completely senesced and were either attached to or detached from the plant. Stem length of the longest stem was measured from the ligule of the youngest fully expanded leaf to the base of the stem at soil level. Leaf area was determined by length and width (at half leaf length) measurements of the youngest fully expanded leaf with a ligule as described by Clifton-Brown and Lewandowski (2000):

LA = 0.74 × LL × LW(1)

where LA is leaf area (cm^2^), LL is the leaf length (cm), and LW is leaf width at half LL (cm).

Harvested plants were separated into leaves, stems, rhizomes, and roots and the final morphological parameters were measured (*n* = 5). Above- and below ground biomass was harvested and fresh weight (FM) was measured, followed by drying at 60 °C until a constant weight was achieved to estimate dry matter (DM).

### 4.3. Stomatal Conductance (g_s_)

Measurements of stomatal conductance (*g*_s_, mmol m^−2^ s^−1^) were performed using a diffusion AP4 porometer (Delta-T Devices Ltd., Cambridge, UK). A single reading was recorded after conductance readings had stabilised for at least three cycles and no more than five cycles.

### 4.4. Relative Water content (RWC)

Relative water content (RWC) indicates the hydration state of the leaf and is a function of the water content of a leaf (*n* = 5) relative to its fully hydrated or fully turgid state and is calculated using the following equation:

RWC = (FM − DM)/(TW − DM)(2)

To measure the fresh matter (FM), screw-cap tubes (2.5 mL) were weighed and numbered. Leaf discs from each plant (days 1–16: *n* = 20; days 17–26: *n* = 15; days 27–37: *n* = 10; and days 38–54: *n* = 5) were harvested in capped tubes and stored on ice until all samples were collected and weighed. The turgid weight (TW) was assessed using rehydrated freshly weighed leaves after floating on distilled water in a Petri dish for 3–4 h. To determine DM the cap was removed, and the samples were dehydrated at 60 °C overnight. After reaching a constant weight, the tubes were sealed, let to cool in room temperature and reweighed. Changes in RWC are proportional to alterations in leaf turgor state; thus, it is considered an indirect measure of change in turgor under certain conditions.

### 4.5. Relative Chlorophyll Content

Relative chlorophyll content was measured according to Stavridou et al. [7] on three leaves per plant and 5 biological replicates per treatment and time point using a SPAD-502 m (Konica Minolta Optics Inc., Osaka, Japan).

### 4.6. In Situ Chlorophyll Fluorescence

The assessment of chlorophyll *a* fluorescence in dark-adapted state was performed on the adaxial leaf surface of the youngest fully expanded leaf with a ligule using a Handy PEA chlorophyll fluorimeter (Hansatech Instruments Ltd., King’s Lynn, UK) after 30 min of dark adaptation. The fluorescence parameters of maximal fluorescence (*F*_m_), minimal fluorescence (*F*_o_), variable fluorescence (*F*_v_), maximal quantum efficiency of PSII photochemistry (*F*_v_/*F*_m_), and the performance index (PI), amongst others, were calculated based on the Strasser et al. (2000, 2004) using the manufacturer’s software (Hansatech Instruments Ltd., King’s Lynn, UK).

The area above the fluorescence curve between *F*_o_ and *F*_m_ is essentially proportional to the pool size of the electron acceptors, Qa, on the reducing side of photosystem II (PSII) and is calculated by the Handy PEA fluorimeter. The area component is an informative parameter highlighting alterations occurred in the shape of the induction kinetic between *F*_o_ and *F*_m_. The hypothesis is that the area should be reduced if proton donation is reduced from lack of H_2_O, and the electron transfer from the reaction centres to the quinone pool is blocked. Using the reduction in the area component may explain any alteration in the shape of the induction kinetics between *F*_o_ and *F*_m_.

### 4.7. Photosynthetic Intercellular-CO_2_ Response Curves

Gas exchange data were used to predict the variables of the von Caemmerer, [101] C4 model and the Excel fitting tool (EFT) [102] was used to derive a set of C4 photosynthetic parameters. The measurements of the response of *A* to the intercellular-CO_2_ (C_i_) were conducted on the fully expanded leaf with a ligule (*n* = 5) using a portable infra-red gas analyser GFS-3000FL (Walz Measurement Instrumentation, Effeltrich, Germany). Prior to the *A*/C_i_ curves, a light response curve was performed to identify the light saturating point of photosynthesis. Measurements of *A* were made starting at photosynthetic active radiation (PAR) of 500, 1000, 1249, 1500, 2000, and 500 μmol m^−2^ s^−1^, while the [CO_2_] was kept at 390 μmol mol^−1^. Leaves were initially dark-adapted (approximately 20 min), so that all the centres of PSII were at an open state and energy dissipation through heat would be minimal and then were placed in the cuvette. After 10 min of complementary dark adaptation a measurement of the *F*_v_/*F*_m_ was recorded. Then the photosynthetic photon flux density (Q) was maintained at 1500 μmol m^−2^ s^−1^ using a chamber integrated red-blue light source. Measurements of *A* initiated at 400 μmol mol^−1^ [CO_2_] surrounding the leaf for 10 min and allowing for the leaf to reach a stable value [103]. The CO_2_ concentrations were modified to change stepwise to the following levels in sequence, 390, 200, 100, 75, 50, 25, 400,600, 800, and 1500 μmol mol^−1^ [CO_2_]. The leaf remained at each CO_2_ level until a stable *A* was determined. The leaf temperature was controlled at 26.4 °C and the vapor pressure deficit (VPD) of the air entering the gas exchange system was 7 Pa kPa^−1^ in average.

The response of *A* to C_i_ at C_i_ < 70 μmol mol^−1^ was used to solve for V*pmax* [101]. The CO_2_-saturated photosynthetic rate (V*pr*) was estimated from the horizontal asymptote of a non-rectangular hyperbolic function for each *A*/C_i_ curve. For each *A*/C_i_ response curve, the carboxylation efficiency (CE) of PEPc was calculated as the slope of the initial linear portion of the curve (C_i_ < 100 μmol mol^−1^), where photosynthesis is controlled by PEP regeneration and/or carboxylation limitation within the bundle-sheath. The operating point of photosynthesis (C_i_, 400) was calculated as the C_i_ that corresponds to a given C_a_ of 400 μmol mol^−1^, fit using a linear regression of the ratio of intercellular to growth CO_2_ (C_i_/C_a_) for each individual leaf [104,105]. The photosynthetic rate where C_i_ = C_a_ (400 μmol mol^−1^) represents the hypothetical scenario in which there is no stomatal limitation to photosynthesis. The percent reduction in photosynthesis due to stomatal limitation (*L*s) was calculated from each replicate *A*/C_i_ curve according to [104] as

*L*s = (*A*_o_ − *A*)/*A*_max_ × 100 (3)

where *A*_o_ is the assimilation rate that would occur if resistance to CO_2_ diffusion in sites of carboxylation were zero (i.e., when C_i_ = C_a_ with C_a_ being the ambient concentration of CO_2_) and *A* is the actual rate at the C_i_ corresponding to the normal C_a_.

For illustrative purposes, mean response curves of *A*/C_i_ were fitted with a non-rectangular hyperbola for all data pooled within each genotype and treatment (Figure S4).

### 4.8. Intrinsic Leaf Water Use Efficiency (WUEi)

Intrinsic leaf water use efficiency (WUEi) was assessed as the ratio of CO_2_ assimilation (*A*) over stomatal conductance (*g*_s_) at photon fluxes of 300 (net irradiance) and 1500 μmol m^−2^ s^−1^ (saturating irradiance) (*n* = 5). The *A*/*g*_s_ is considered a more realistic and comparable between studies parameter, as it is not affected by alterations in leaf to air VPD in the leaf chamber [106,107].

### 4.9. Proline Content and Lipid Peroxidation

For the analyses of proline content and lipid peroxidation, sampling of leaves was performed on 5 biological replicates. Proline (μmol g^−1^ FW) cold extraction procedure was performed according to [108] by mixing 20 mg of leaf fresh weight (FW) aliquots with 400 μL of ethanol: water (40:60 *v/v*). Proline content was measured based on the method of Carillo et al. [109]. Proline content was measured spectrophotometrically at 520 nm with a micro-plate reader (μQuant; Bio-Tek Instruments, Winooski, VT, USA) using KC4 software (v. 3.3; Bio-Tek) using the method of Carillo et al. [109] from three biological and three technical replicates per treatment.

Lipid peroxidation was assessed from the total content of 2-thiobarbituric acid reactive substances (TBARS) expressed as equivalents of malondialdehyde (MDA), which has been extensively used as a biomarker for lipid peroxidation [110] using the method of [111] with the following modifications: ground leaf powder (0.25 g) was homogenised in 1 mL of 0.1% (*w/v*) trichloroacetic acid (TCA) solution and centrifuged at 12,000× *g* for 10 min. The supernatant was added to 1 mL of 0.5% (*w/v*) thiobarbituric acid (TBA) in 20% TCA. A 30 min incubation of the mixture at 95 °C was performed and samples were, placed in an ice bath to stop the reaction and were briefly vortexed. Aliquots of 200 μL from each sample were placed in triplicate in a 96-well plate. The absorbance of the supernatant was measured at 532 nm and 600 nm using a micro-plate reader (μQuant; Bio-Tek Instruments, Winooski, VT, USA) using KC4 software (v. 3.3; Bio-Tek). The MDA-TBA complex (red pigment) was assessed according to Equation (4) [112]:

MDA equivalents (nmol g^−1^) = [(*A*532 − *A*600) *ε* 1000 *V*]/FW (4)

where *A*532 and *A*600 (non-specific absorption) are the absorbances at 532 nm and 600 nm, respectively; the excitation coefficient, *ε* = 155 mM cm^−1^; *V* is the volume of the extract (mL); and FW is the fresh weight of each sample (g).

### 4.10. Ash Content

The ash content (%) was determined as previously described in [7]. Ground leaf sample (1 g) (*n* = 5) was initially dried overnight at 100 °C in previously weighed beakers (25 mL). The samples were placed into desiccators to cool and were weighed. Following, the samples were placed in a muffle furnace at 550 °C for 16 h and in an oven at 100 °C to lower the sample temperature and the ash was weighed after 30 min. The ash content (%) in each sample was calculated according to Equation (5):

%Ash (dried basis) = % Mass of the ash sample (g)/original mass of the dried sample (g)(5)

### 4.11. Elemental Content Analysis

For the analysis of the elemental content, oven dried at 60 °C and ground leaf (*n* = 3; at four time points), stem and rhizome (*n* = 5; at harvest) samples were sent to NRM laboratories© (Bracknell, UK). Plant tissue analysis for determination of total elements was performed using microwave digestion. The sample was digested in concentrated nitric acid at high temperature and pressure, to avoid the development of strong oxidising agents that would destroy organic matter and break down the mineral matrix of the sample. The total elements of potassium, magnesium, calcium, sulphur, sodium, iron, aluminium, and titanium in solution were then assessed by Inductively Coupled Plasma Optical Emission Spectroscopy (ICP-OES) (The Analysis of Agricultural Materials, MAFF Reference Book RB427, ISBN 0 11 242762 6). Chloride was extracted from the dried plant material with deionised water and determined by ion chromatography. Quantification was performed by peak area or height. The method of known additions was used to resolve uncertainties of identification or quantification. All recovery data were between 90 and 100%. The lowest detectable concentration of an anion was determined as a function of the sample size and the conductivity scale used. Generally, this is 0.01% for chloride. (Standard Methods for the Examination of Water and Wastewater 1985, 16th Edition). For the silicon (Si) assay the acid-insoluble ash method was used. Residual dry matter was assessed gravimetrically as the residue remaining after drying at 102 °C for 16 h. Acid-insoluble ash is the insoluble residue remaining after the sample was ignited in a muffle furnace at 450 °C and the ash treated with hydrochloric acid. The insoluble residue after acid treatment was filtered, washed, and ignited at 600 °C. (MAFF/ADAS The Analysis of Agricultural Materials. Reference Book 427. H.M.S.O, London, UK; 1986: 16–17).

The base to acid ratio (R_b/a_) (Equation (6)) is often used to determine the fouling tendency of a fuel ash (Baxter et al., 2012).

R_b/a_ = (Fe_2_O_3_ + CaO + MgO + K_2_O + Na_2_O)/(SiO_2_ + TiO_2_ + Al_2_O_3_) (6)

The R_b/a_ and % Base indices were calculated from the estimated oxides of each compound in the biomass as an approximation to their content in the ash. The weight concentration of an element to its oxide is the ratio of the oxide molecular weight to the element atomic weight. The corrosion potential index 2S/Cl was the estimated molar ratio of S and Cl, which can be calculated as the ratio of the element weight to its molecular weight.

### 4.12. Statistical Analyses

All statistical analyses were performed using R (R Core Team, 2016). The effects of the different salinity treatments on the morphophysiological, photophysiological, and biochemical parameters compared to the control plants were assessed using one-way ANOVAs, whereas the time course measurements using two-way ANOVAs (salinity as between subjects’ and days as within subjects’ effects) with the afex packages [113]. Data were tested for normality (Shapiro test) and transformations were attempted when normality failed. For the two-way ANOVA data were also tested with Mauchly’s test for sphericity and if the assumption of sphericity was violated the corresponding Greenhouse–Geisser corrections were performed. If significant differences were observed among treatments, then the Tukey’s HSD post-hoc test was performed to determine specific treatment, time point, and interaction differences using the Agricolae package [114].

## Figures and Tables

**Table 1:** Significant effects of treatment, the different harvest days and their interaction based on the ANOVA of morphological and physiological parameters of *M. × giganteus* growing in different NaCl concentrations (0, 5.44, and 19.97 dS m^−1^).

Parameters	Treatment	Harvest day	Interaction
Final height	<0.05	ns	<0.1
Leaf area (LA)	<0.05	ns	ns
Total leaves	<0.05	<0.1	ns
Fresh matter (FM) above	<0.001	<0.05	ns
FM leaves	<0.001	ns	<0.05
FM stems	<0.001	<0.01	ns
FM below	<0.001	<0.05	<0.05
FM rhizome	<0.001	<0.001	<0.001
FM roots	<0.001	<0.05	ns
FM total	<0.001	<0.001	<0.001
Dry matter (DM) above	<0.001	<0.001	<0.001
DM leaves	<0.001	<0.001	ns
DM stems	<0.001	<0.001	<0.001
DM below	<0.001	<0.001	<0.001
DM rhizome	<0.001	<0.001	<0.001
DM roots	<0.01	<0.001	<0.001
DM total	<0.001	<0.001	<0.001
Relative Water Content (RWC)	<0.01	<0.1	<0.1
%Electrolyte Leakage (EL)	<0.001	<0.001	<0.001
Malondialdehyde (MDA)	<0.001	<0.001	<0.05
Proline	<0.05	ns	<0.001

**Table 2:** Tukey’s honestly significant difference (T_HSD_) post-hoc test for the interaction effects of treatment and harvest point (Days) on the DM of the above- and belowground biomass, leaves, stems, and roots and rhizomes at 0, 5.44, and 19.97 dS m^−1^ NaCl. Different lowercase letters indicate significant differences between treatments (BT) for each harvest day; uppercase letters differences within treatment (WT) for each across harvest days at *p* < 0.05; ns indicate no significant differences. Data are mean ± Standard Error.

Days	NaCl	Above DM ± SE	T_HSD_ BT	T_HSD_ WT	Leaves DM ± SE	T_HSD_ BT	T_HSD_ WT	Stems DM ± SE	T_HSD_ BT	T_HSD_ WT	Below DM ± SE	T_HSD_ BT	T_HSD_ WT	Rhizome DM ± SE	T_HSD_ BT	T_HSD_ WT	Roots DM ± SE	T_HSD_ BT	T_HSD_ WT
19	0	46.7 ± 2.1	a	B	22.4 ± 1.2	a	B	24.5 ± 0.9	a	C	27.6 ± 1.0	a	B	16.4 ± 0.7	a	ns	11.2 ± 0.6	a	C
	5.44	42.7 ± 3.2	ab	ns	19.8 ± 1.3	a	B	23.0 ± 2.1	a	B	29.3 ± 2.7	a	C	17.3 ± 2.1	a	A	12.0 ± 0.9	a	C
	19.97	33.5 ± 2.7	b	ns	14.8 ± 1.2	b	ns	18.7 ± 1.5	a	ns	30.3 ± 2.3	a	ns	20.9 ± 1.6	a	A	9.4 ± 0.6	a	B
32	0	53.1 ± 1.6	a	B	22.7 ± 0.6	a	B	30.4 ± 1.5	a	BC	42.6 ± 5.4	a	B	18.7 ± 3.6	a	ns	23.9 ± 2.8	a	BC
	5.44	41.8 ± 3.2	b	ns	17.9 ± 1.3	b	B	23.9 ± 2.0	ab	B	31.5 ± 4.4	a	BC	9.5 ± 0.9	b	B	22.1 ± 3.5	a	B
	19.97	35.7 ± 2.8	b	ns	15.2 ± 1.1	b	ns	20.5 ± 1.8	b	ns	32.9 ± 3.1	a	ns	10.9 ± 0.7	ab	B	22.0 ± 2.5	a	A
46	0	61.8 ± 6.2	a	B	24.6 ± 2.6	a	B	37.3 ± 3.7	a	B	46.3 ± 3.5	a	B	13.4 ± 1.5	a	ns	32.9 ± 3.4	a	BC
	5.44	50.2 ± 2.9	b	ns	20.9 ± 1.1	a	B	29.3 ± 1.9	ab	B	45.4 ± 4.7	ab	AB	12.1 ± 1.5	ab	AB	33.3 ± 2.8	a	A
	19.97	38.5 ± 4.0	b	ns	18.1 ± 1.6	a	ns	20.5 ± 2.4	b	ns	31.9 ± 3.6	b	ns	8.4 ± 0.8	b	B	23.5 ± 3.3	a	A
54	0	88.9 ± 4.0	a	A	33.8 ± 1.5	a	A	55.2 ± 2.7	a	A	68.8 ± 6.7	a	A	20.9 ± 1.1	a	ns	47.9 ± 7.1	a	A
	5.44	65.4 ± 0.6	b	ns	25.9 ± 0.6	b	A	39.5 ± 1.1	b	A	56.7 ± 2.4	a	A	15.6 ± 1.4	b	AB	41.1 ± 1.2	a	A
	19.97	41.1 ± 3.3	c	ns	19.7 ± 1.8	c	ns	21.4 ± 1.8	c	ns	27.6 ± 2.6	b	ns	9.5 ± 1.2	c	B	18.1 ± 1.5	b	A

**Table 3:** Tukey *HSD* (T_HSD_) post-hoc test for the effects of treatment and harvest day on proline (μmol g^−1^ FW) at 0, 5.44, and 19.97 dS m^−1^ NaCl. Different lowercase letters indicate significant differences between treatments (BT) for each time point (Week: 1, 3, 5, and 7); uppercase letters differences within treatment (WT) across harvest days at *p* < 0.05; ns indicate no significant differences. Data are mean ± Standard Error (*n* = 5).

Harvest Day	NaCl	Proline ± SE	T_HSD_ BT	T_HSD_ WT
19	0	0.017 ± 0.001	a	A
	5.44	0.015 ± 0.001	a	ns
	19.97	0.019 ± 0.003	a	B
32	0	0.007 ± 0.001	b	B
	5.44	0.007 ± 0.001	b	ns
	19.97	0.17 ± 0.061	a	A
46	0	0.007 ± 0.001	b	B
	5.44	0.008 ± 0.001	b	ns
	19.97	0.238 ± 0.036	a	A
54	0	0.006 ± 0.001	c	B
	5.44	0.016 ± 0.005	b	ns
	19.97	0.238 ± 0.036	a	A

**Table 4:** Significant effects of treatment, tissue type, harvest day, and their interactions based on the ANOVA of morphological and physiological parameters of *M. × giganteus* growing in different NaCl concentrations (0, 5.44, and 19.97 dS m^−1^).

Effects	Ash Content
Treatment	<0.001
Harvest day	<0.1
Tissue	<0.001
Tissue * Treatment	<0.001
Treatment * Harvest day	<0.001

**Table 5:** Tukey *HSD* (T_HSD_) post-hoc test for the effects of treatment on the total element content for K, Na, Cl, Ca, Mg, S, and Si of *M. × giganteus* leaves, stems, and rhizomes at 0, 5.44, and 19.97 dS m^−1^ NaCl on harvest day 54. Different lowercase letters indicate significant differences between treatments (BT) for each tissue type; uppercase letters differences within treatment (WT) between tissue types at *p* < 0.05; ns indicate no significant differences. Data are mean ± Standard Error (Leaves: *n* = 3; Stems and Rhizomes: *n* = 5).

Element	NaCl	Leaves (mg kg^−1^)	T_HSD_ BT	T_HSD_ WT	Stems (mg kg^−1^)	T_HSD_ BT	T_HSD_ WT	Rhizome (mg kg^−1^)	T_HSD_ BT	T_HSD_ WT
K	0	4851 ± 280.6	b	B	4717 ± 377	b	B	13,904 ± 1082	ns	A
	5.44	5639 ± 366.6	b	B	7909 ± 1865	ab	B	13,262.4 ± 299	ns	A
	19.97	14,043± 1851	a	A	11,959 ± 2662	a	A	15,636.2 ± 1742	ns	A
Na	0	67.6 ± 24.7	b	ns	723 ± 644	c	ns	235.2 ± 78.7	c	ns
	5.44	907 ± 276	b	C	2908 ± 435	b	B	4842 ± 135.6	b	A
	19.97	22,891 ± 2625	a	A	12,126 ± 846	a	B	9871 ± 377	a	B
Cl	0	3866 ± 868	b	ns	16,260 ± 8452	ns	ns	2520 ± 165	c	ns
	5.44	10,333 ± 3788	ab	B	37,020 ± 9488	ns	A	9560 ± 409	b	B
	19.97	35,433 ± 9249	a	A	29,240 ± 2734	ns	A	15,880 ± 603	a	B
Ca	0	6488 ± 602	b	A	1309 ± 84	b	B	810 ± 376	ns	B
	5.44	9298 ± 417	a	A	1622 ± 32	b	B	379 ± 8.71	ns	C
	19.97	9679 ± 730	a	A	1962 ± 137	a	B	633.8 ± 113	ns	C
Mg	0	5685 ± 471.6	ns	A	2407 ± 133	ns	B	738 ± 39.8	b	C
	5.44	7391 ± 676	ns	A	2514 ± 165	ns	B	752.8 ± 40.8	b	C
	19.97	5848 ± 801	ns	A	3090 ± 334	ns	B	1026 ± 102.8	a	C
S	0	875 ± 43.7	ns	B	733 ± 57	b	B	1207 ± 49.7	b	A
	5.44	740 ± 18	ns	B	697 ± 43	b	B	1106 ± 46.9	b	A
	19.97	924 ± 86.9	ns	B	1043 ± 95.8	a	B	1478 ± 61.7	a	A
Si	0	5566 ± 433	b	A	1080 ± 193	b	B	-	-	-
	5.44	6700 ± 737	ab	A	1200 ± 130	b	B	-	-	-
	19.97	8500 ± 503	a	A	4100 ± 892	a	B	-	-	-

**Table 6:** Tukey *HSD* (T_HSD_) post-hoc test for the effects of treatment on the total element content for K/Na, Ca/Na, Ca/K, and Si/K of *M. × giganteus* leaves, stems and rhizomes at 0, 5.44, and 19.97 dS m^−1^ NaCl on harvest day 54. Different lowercase letters indicate significant differences between treatments (BT) for each tissue type; uppercase letters differences within treatment (WT) between tissue types at *p* < 0.05; ns indicate no significant differences. Data are mean ± Standard Error (Leaves: *n* = 3; Stems and Rhizomes: *n* = 5).

Element	NaCl	Leaves (mg kg^−1^)	T_HSD_ BT	T_HSD_ WT	Stems (mg kg^−1^)	T_HSD_ BT	T_HSD_ WT	Rhizome (mg kg^−1^)	T_HSD_ BT	T_HSD_ WT
K/Na	0	86.8 ± 20.8	a	ns	55.8 ± 19.6	a	ns	74.9 ± 17.3	a	ns
	5.44	7.43 ± 1.99	b	A	2.66 ± 0.29	b	B	2.75 ± 0.15	b	B
	19.97	0.62 ± 0.10	c	B	0.97 ± 0.18	c	AB	1.58 ± 0.18	c	A
Ca/Na	0	120 ± 34.2	a	ns	14.43 ± 4.28	a	ns	3.13 ± 0.37	a	ns
	5.44	11.8 ± 2.7	b	A	0.60 ± 0.09	b	B	0.08±0.004	b	C
	19.97	0.43±0.1	c	A	0.17±0.01	c	B	0.064±0.01	b	C
Ca/K	0	1.34 ± 0.15	ab	A	0.28 ± 0.02	ns	B	0.09 ± 0.06	ns	C
	5.44	1.67 ± 0.17	a	A	0.24 ± 0.03	ns	B	0.03 ± 0.0004	ns	C
	19.97	0.73 ± 0.17	b	A	0.19 ± 0.03	ns	B	0.045 ± 0.01	ns	C
Si/K	0	1.15 ± 0.06	a	A	0.24 ± 0.05	ns	B	-	-	-
	5.44	1.21 ± 0.18	a	A	0.18 ± 0.04	ns	B	-	-	-
	19.97	0.62 ± 0.07	b	A	0.41 ± 0.14	ns	B	-	-	-

**Table 7:** Tukey *HSD* (T_HSD_) post-hoc test for the effects of treatment on the combustion indices base to acid ratio (Rb/a) and Base (%) of *M. × giganteus* leaves and stems at 0, 5.44, and 19.97 dS m^−1^ NaCl on harvest day 54. Different lowercase letters indicate significant differences between treatments (BT) for each tissue type; uppercase letters differences within treatment (WT) between tissue types at *p* < 0.05; ns indicate non- significant differences. Data are mean ± Standard Error (Leaves: *n* = 3; Stems: *n* = 5).

Index	NaCl	Leaves	T_HSD_ BT	T_HSD_ WT	Stems	T_HSD_ BT	T_HSD_ WT
Rb/a	0	2.59 ± 0.24	b	B	10.2 ± 3.08	ab	A
	5.44	2.95 ± 0.26	b	B	14.1 ± 4.08	a	A
	19.97	6.52 ± 0.061	a	A	6.61 ± 1.13	b	A
Base (%)	0	3.0 ± 0.18	c	A	1.92 ± 0.25	c	B
	5.44	4.14 ± 0.1	b	A	3.33 ± 0.61	b	A
	19.97	11.88 ± 0.76	a	A	6.98 ± 0.93	a	B

## Supplementary Materials

The following are available online at https://www.mdpi.com/2223-7747/9/10/1266/s1, Figure S1: Electrical conductivity of the substrate of *M. × giganteus* at 0, 5.44, 19.97 dS m^−1^ over a period of 54 days. Figure S2: Representative leaves with fully expanded ligule of *M.x giganteus* on day 45 at 0, 5.44, 19.97 dS m^−1^ NaCl. Figure S3: Leaf area (LA; cm^2^) of *M. × giganteus* at 0, 5.44, 19.97 dS m^−1^ over a period of 54 days. Figure S4: A/Ci curves. Measured *A*/Ci curve (Left) and modelled *A*/Ci curve derived from a fitted non-rectangular hyperbola (Right) of *M. × giganteus* at 0, 5.44, 19.97 dS m^−1^ over four time points (Weeks: 1, 3, 5 and 7). Figure S5: Total element contents for K, Na, Cl, Ca, Mg, S and Si of *M. × giganteus* leaves (green bars), stems (light blown bars) and rhizomes (dark brown bars) at 0, 5.44, 19.97 dS m^−1^ NaCl on the final harvest day 54., Table S1: Tukey HSD (T_HSD_) post-hoc test for the effects of treatment and harvest day on the FM of the above and below biomass, leaves stems, roots and rhizomes at 0, 5.44, 19.97 dS m^−1^ NaCl. Table S2: Significant effects of treatment, time and their interaction based on the ANOVA of morphological and physiological parameters of *M. × giganteus* growing in different NaCl concentrations (0, 5.44 and 19.97 dS m^−1^). Table S3: Tukey HSD (T_HSD_) *post-hoc* test for the effects of treatment and time point on the model derived parameters from the fitted A/Ci curves (A_max_, CE, V_pmax_, *L*s, WUEi) at 0, 5.44, 19.97 dS m^−1^ NaCl. Table S4: Table S5. Significant effects of treatment, different harvest days and their interaction based on the ANOVA of total elemental content in *M. × giganteus* leaves of plants growing in different NaCl concentrations (0, 5.44 and 19.97 dS m^−1^). Table S6: Tukey HSD (T_HSD_) post-hoc test for the effects of treatment and harvest day on the total element content for K, Na, Cl, Ca, Mg, S, Si and Ti of *M. × giganteus* leaves at 0, 5.44, 19.97 dS m^−1^ NaCl on harvest days 19, 32, 46 and 54. Table S7: Tukey HSD (T_HSD_) post-hoc test for the effects of treatment and harvest day on combustion indices base to acid ratio (Rb/a) and Base (%) of *M. × giganteus* leaves at 0, 5.44, 19.97 dS m^−1^ NaCl on harvest days 19, 32, 46 and 54.
