# Supplementary material for: The Effects of Moderate and Severe Salinity on Composition and Physiology in the Biomass Crop Miscanthus × giganteus
Source: Plants (Basel). 2020 Sep 25;9(10):1266. doi: 10.3390/plants9101266 (PMC7600718; doi:10.3390/plants9101266)
Supplement: Supplementary file 1 [file plants-09-01266-s001.pdf]

Supplementary material

The effects of moderate and severe salinity on composition and physiology in the biomass crop *Miscanthus* × *giganteus*

Evangelia Stavridou [1,2], Richard J. Webster [1,3] and Paul R. H. Robson [1,*]

[1] Institute of Biological, Environmental and Rural Sciences, Aberystwyth University, Aberystwyth SY23 3EE, UK; ; estavrid@certh.gr (E.S), R.J.Webster1@ljmu.ac.uk (R.J.W)
[2] Institute of Applied Biosciences, Centre for Research and Technology-Hellas, Thessaloniki GR570 01, Greece
[3] School of Biological and Environmental Sciences,, Liverpool John Moores University, Liverpool L3 3AF, UK
* Correspondence: ppr@aber.ac.uk

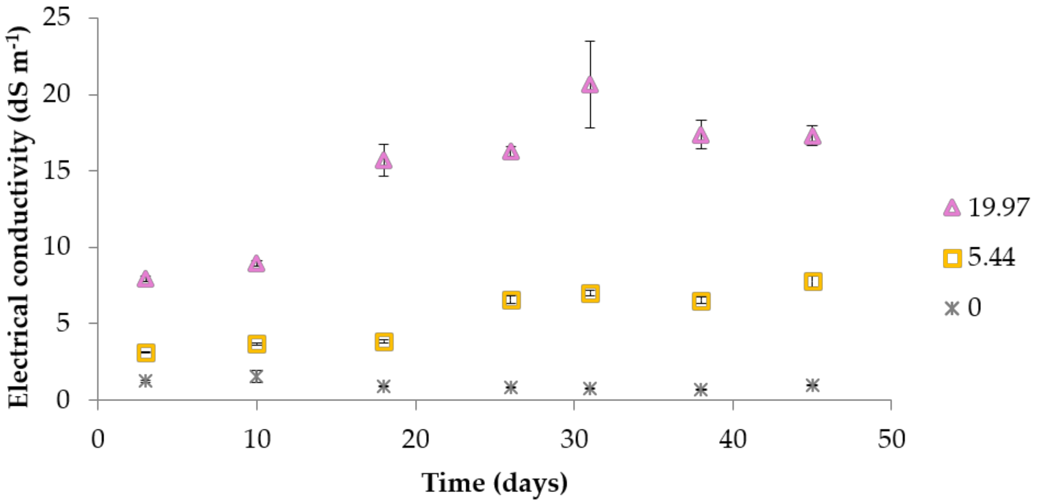

Figure S1. Electrical conductivity of the substrate of *M.* × *giganteus* at 0, 5.44, 19.97 dS m^{-1} over a period of 54 days. Data are mean ± Standard Error (days 1-16: n=20; days 17-26: n=15; days 27-37: n=10; days 38-54: n=5).

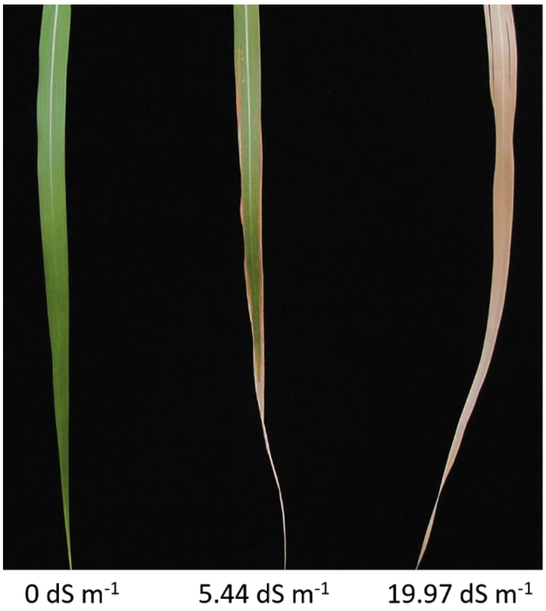

Figure S2. Representative leaves with fully expanded ligule of *M.× giganteus* on day 45 at 0, 5.44, 19.97 dS m^{-1} NaCl. The leaf at the 19.97 dS m^{-1} was over 2/3 senesced.

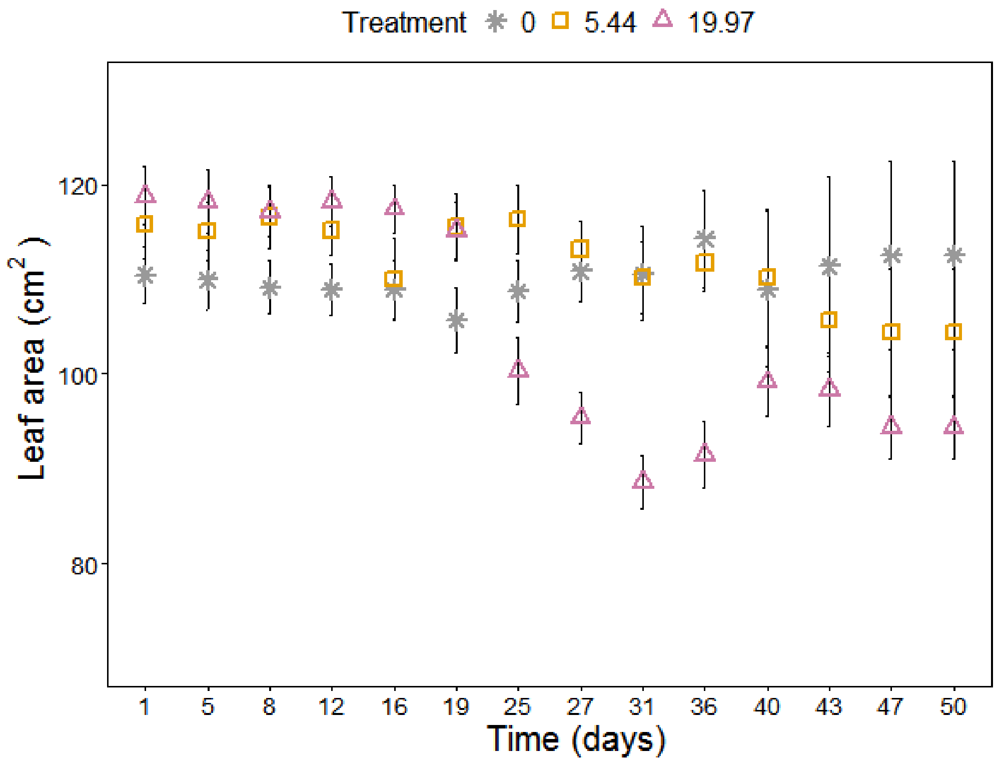

Figure S3. Leaf area (LA; cm^2) of *M.* × *giganteus* at 0, 5.44, 19.97 dS m^{-1} over a period of 54 days. Data are mean ± Standard Error (days 1-16: n=20; days 17-26: n=15; days 27-37: n=10; days 38-54: n=5).

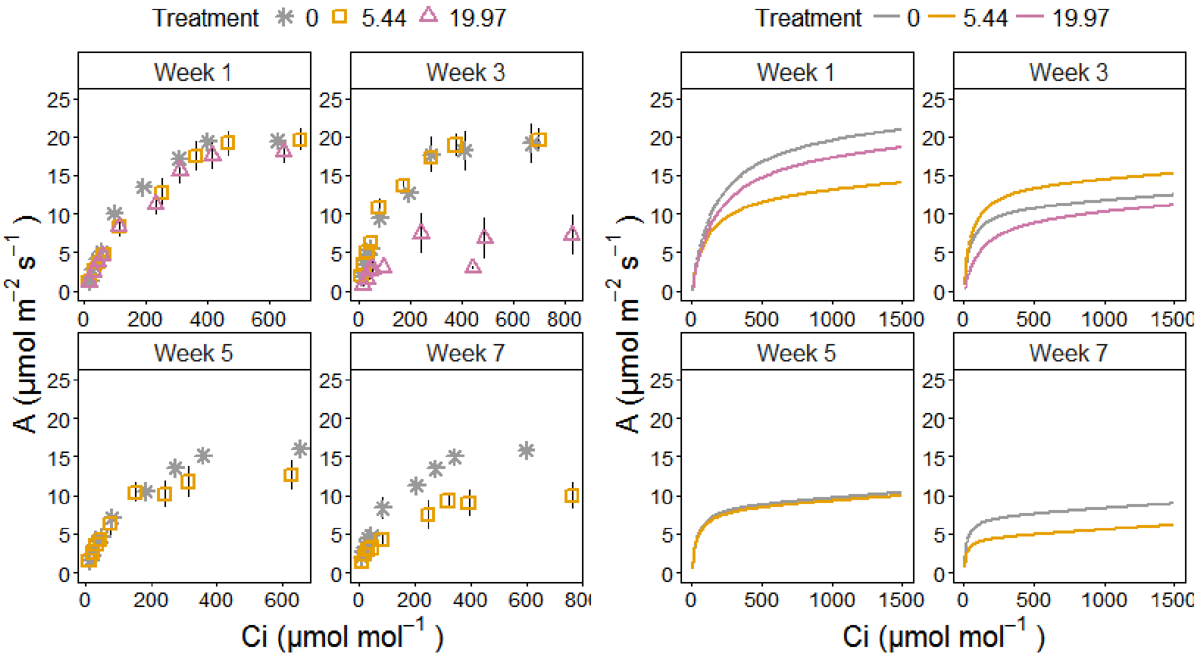

Figure S4. A/Ci curves. Measured A/C_i curve (Left) and modelled A/C_i curve derived from a fitted non-rectangular hyperbola (Right) of *M.* × *giganteus* at 0, 5.44, 19.97 dS m^{-1} over four time points (Weeks: 1, 3, 5 and 7). Data are mean ± Standard Error (n=5).

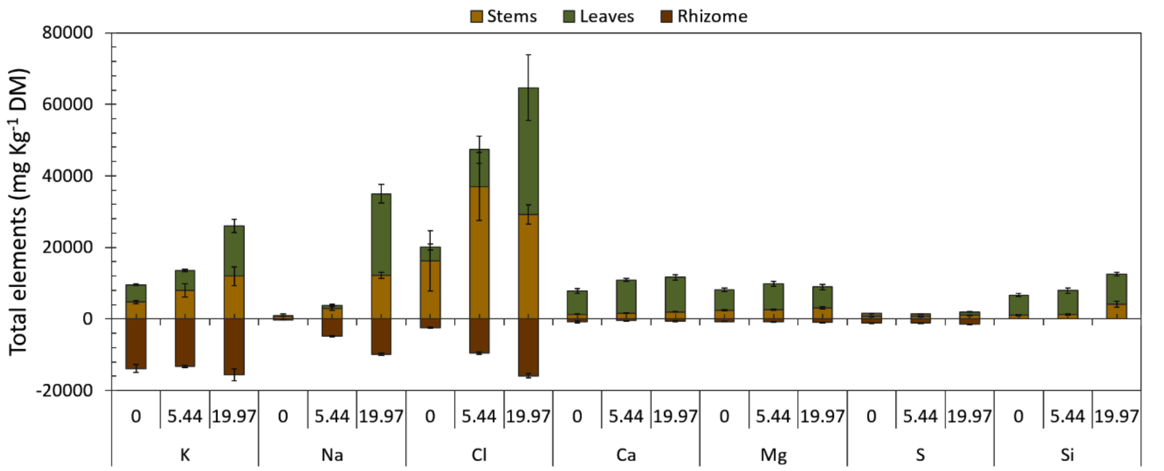

Figure S5. Total element contents for K, Na, Cl, Ca, Mg, S and Si of *M.* × *giganteus* leaves (green bars), stems (light blown bars) and rhizomes (dark brown bars) at 0, 5.44, 19.97 dS m^{-1} NaCl on the final harvest day 54. Data are mean ± Standard Error (Leaves: n=3; Stems and Rhizomes: n=5).

Table S1. Tukey HSD (THSD) *post-hoc* test for the effects of treatment and harvest day on the FM of the above and below biomass, leaves stems, roots and rhizomes at 0, 5.44, 19.97 dS m^{-1} NaCl. Different lowercase letters indicate significant differences between treatments (BT) for each harvest day; uppercase letters differences within treatment (WT) for each across harvest days at p<0.05; ns indicate no significant

Harvest day	NaCl	Above FM±SE	THSD BT	THSD WT	Leaves FM±SE	THSD BT	THSD WT	Stems FM±SE	THSD BT	THSD WT	Below FM±SE	THSD BT	THSD WT	Rhizomes FM±SE	THSD BT	THSD WT	Roots FM±SE	THSD BT	THSD WT
19	0	160.5±9.5	a	B	59.4±2.9	a	B	101.1±6.78	a	B	164.9±11.1	a	B	84.3±7.9	a	B	72±3.6	a	AB
	5.44	130.0±9.0	a	ns	48.6±3.1	b	ns	81.5±6.10	a	ns	147.6±15.3	a	ns	66.4±8.3	a	B	64.3±5.9	a	ns
	19.97	85.7±6.9	b	ns	30.2±2.3	c	ns	55.6±4.73	b	ns	129.2±11.6	a	ns	71.8±5.4	a	ns	44.9±3.2	b	ns
32	0	177.4±9.6	a	B	68.0±3.0	a	AB	109.5±7.07	a	B	231.8±31.3	a	AB	116.5±13.2	a	B	115±21.5	a	A
	5.44	132.7±11.6	b	ns	49.0±4.1	b	ns	83.7±7.69	ab	ns	152.9±19.4	ab	ns	95.6±15.2	a	AB	57.3±5.1	b	ns
	19.97	83.3±8.0	c	ns	24.6±2.3	c	ns	58.8±5.87	b	ns	126.6±8.1	b	ns	71.4±6.1	a	ns	55.3±3.6	b	ns
46	0	170.7±10.4	a	B	59.1±3.6	a	B	111.7±6.99	a	A	209.3±10.9	a	AB	124.5±3.89	a	B	67.6±6.3	a	B
	5.44	133.7±7.3	b	ns	48.2±1.8	b	ns	85.6±5.62	ab	ns	182.7±18.8	ab	ns	108.9±10.4	a	AB	62.2±8.6	ab	ns
	19.97	89.2±10.7	c	ns	24.1±2.2	c	ns	65.2±8.51	b	ns	127.9±15	b	ns	70.1±10.0	b	ns	42.5±4.7	b	ns
54	0	215.7±7.1	a	A	73.7±2.6	a	A	142.1±5.76	a	AB	289.4±23.3	a	A	187.2±22.2	a	A	102.3±4.6	a	AB
	5.44	138.6±3.9	b	ns	46.4±1.7	b	ns	92.3±2.46	b	ns	190.6±6.5	b	ns	116.8±2.8	b	A	73.8±6.3	b	ns
	19.97	92.0±9.7	c	ns	24.6±2.8	c	ns	67.4±7.24	c	ns	117.7±11.1	c	ns	66.1±5.7	c	ns	51.7±5.7	c	ns

Table S2. Significant effects of treatment, time and their interaction based on the ANOVA of morphological and physiological parameters of *M.* × *giganteus* growing in different NaCl concentrations (0, 5.44 and 19.97 dS m^{-1}).

Parameters	Treatment	Day	Interaction
F_v/F_m	<0.001	<0.001	<0.001
PI	<0.001	<0.001	<0.001
g_s	<0.001	<0.001	<0.001
SPAD	<0.001	<0.001	<0.001
CE_{amb}	<0.001	ns	<0.05
A_{max}	<0.001	<0.001	<0.001
ω	ns	<0.001	ns
CO_2 Compensation Point	<0.05	<0.01	ns
Ls	<0.001	<0.01	ns
Vpmax	ns	<0.01	ns
Kp	ns	<0.01	ns
WUEi	<0.001	<0.01	<0.001
Rd	ns	ns	ns
Height	ns	<0.001	<0.001
Number of senesced leaves	<0.05	<0.001	<0.001
Leaf area (LA)	ns	<0.001	<0.001

Table S3. Tukey HSD (T_{HSD}) *post-hoc* test for the effects of treatment and time point on the model derived parameters from the fitted A/Ci curves (A_{max}, CE, V_{pmax}, *L*s, WUEi) at 0, 5.44, 19.97 dS m^{-1} NaCl. Different lowercase letters indicate significant differences between treatments (BT) for each time point (Week: 1, 3, 5 and 7); uppercase letters differences within treatment (WT) for each across harvest days at *p*<0.05; ns indicate no significant differences. Data are mean ± Standard Error (n=5).

Week	NaCl	A_{max} ±SE	T_{HSD} BT	T_{HSD} WT	CE ±SE	T_{HSD} BT	T_{HSD} WT	Vpmax ±SE	T_{HSD} BT	T_{HSD} WT	L_s ±SE	T_{HSD} BT	T_{HSD} WT	WUEi ±SE	T_{HSD} BT	T_{HSD} WT
	0	22.5±1.58	ns	ns	0.12±0.03	a	ns	28.6±7.79	a	ns	22.4±2.28	a	ns	0.35±0.02	a	B
1	5.44	23±0.63	ns	A	0.09±0.01	a	ns	16.6±6.15	a	ns	17.9±6.55	a	ns	0.31±0.06	a	B
	19.97	22.1±1.92	ns	A	0.13±0.01	a	A	25.8±4.55	a	A	20.3±3.06	a	A	0.34±0.053	a	ns
	0	23.8±4.15	a	ns	0.16±0.01	a	ns	19.9±7.52	a	ns	18.5±2.91	a	ns	0.45±0.014	a	A
3	5.44	22.5±1.82	a	A	0.19±0.02	a	ns	33.3±14.13	a	ns	16.7±1.13	a	ns	0.43±0.02	a	AB
	19.97	8.3±3.13	b	B	0.06±0.03	b	B	6.7±2.96	a	B	7.3±2.56	b	B	0.35±0.08	a	ns
	0	20.8±1.74	a	ns	0.15±0.02	a	ns	13.3±3.28	a	ns	20.7±3.69	a	ns	0.46±0.03	a	A
5	5.44	14.2±2.17	b	B	0.14±0.03	a	ns	18.6±9.28	a	ns	20.2±3.19	a	ns	0.48±0.03	a	A
	19.97	-	-	-	-	-	-	-	-	-	-	-	-	-	-	-
	0	19.3±1.31	a	ns	0.17±0.04	a	ns	13.4±4.47	a	ns	16.7±2.47	a	ns	0.49±0.01	a	A
7	5.44	11.1±2.2	b	B	0.12±0.03	a	ns	6.1±1.45	ab	ns	11.7±2.78	a	ns	0.39±0.02	b	AB
	19.97	-	-	-	-	-	-	-	-	-	-	-	-	-	-	-

Table S4. Significant effects of treatment, tissue type and their interaction based on the ANOVA of total elemental content of *M.* × *giganteus* growing in different NaCl concentrations (0, 5.44 and 19.97 dS m^{-1}).

Parameters	Treatment	Tissue	Interaction
Total K	<0.001	<0.001	ns
Total Na	<0.001	ns	<0.001
Water soluble Cl	<0.01	<0.001	ns
Total Ca	<0.001	<0.01	<0.05
Total Mg	<0.001	<0.05	<0.1
Total S	<0.001	<0.001	ns
Total Fe	ns	<0.001	ns
Si	<0.01	<0.001	ns
Ca/K	<0.05	<0.001	<0.1
Si/K	<0.1	<0.001	<0.001

Table S5. Significant effects of treatment, different harvest days and their interaction based on the ANOVA of total elemental content in *M.* × *giganteus* leaves of plants growing in different NaCl concentrations (0, 5.44 and 19.97 dS m^{-1}).

Parameters	Treatment	Harvest day	Interaction
Total K	<0.001	<0.001	ns
Total Na	<0.001	<0.001	<0.001
Water soluble Cl	<0.001	<0.1	ns
Total Ca	<0.1	<0.05	ns
Total Mg	ns	<0.05	ns
Total S	<0.01	<0.001	<0.01
Total Fe	ns	<0.05	ns
Si	<0.001	<0.1	ns
Al	ns	<0.05	ns
Ti	<0.01	<0.1	ns
Ca/K	<0.01	<0.001	ns
Si/K	<0.01	<0.05	<0.1

Table S6. Tukey HSD (THSD) post-hoc test for the effects of treatment and harvest day on the total element content for K, Na, Cl, Ca, Mg, S, Si and Ti of *M.* × *giganteus* leaves at 0, 5.44, 19.97 dS m^{-1} NaCl on harvest days 19, 32, 46 and 54. Different lowercase letters indicate significant differences between treatments (BT) for each harvest day; uppercase letters differences within treatment (WT) between harvest days at $p<0.05$; ns indicate no significant differences. Data are mean ± Standard Error (n=3).

Total element	Harvest days	19			32			46			54		
	NaCl	mg Kg^{-1} ± SE	THSD BT	THSD WT	mg Kg^{-1} ± SE	THSD BT	THSD WT	mg Kg^{-1} ± SE	THSD BT	THSD WT	mg Kg^{-1} ± SE	THSD BT	THSD WT
Cl	0	2000±115	c	B	3900±305	c	A	2800±200	c	AB	3866±868	b	A
	5.44	9600±153	b	ns	13066±517	b	ns	14133±1304	b	ns	10333±3788	ab	ns
	19.97	20066±2085	a	ns	35133±4148	a	ns	42866±1617	a	ns	35433±9249	a	ns
K	0	8701±455	b	A	8856±890	b	A	4658±603	c	B	4851±281	b	B
	5.44	10537±2092	ab	AB	11485±975	b	A	8360±174	b	AB	5639±366	b	B
	19.97	15941±1123	a	ns	15727±880	a	ns	10770±657	a	ns	14043±1851	a	ns
Na	0	32±1.36	b	ns	39±15	b	ns	33±5.69	c	ns	67.6±24.7	c	ns
	5.44	127±78	b	B	102±18	b	B	87±7.28	b	B	907±276	b	A
	19.97	2430±765	a	B	13227±2706	a	A	20738±1097	a	A	22891±2625	a	A
S	0	1829±200	a	A	1626±238	a	A	1181±135	ns	AB	876±43.7	ns	B
	5.44	1302±59.4	b	A	1025±46.7	ab	AB	864±50.8	ns	BC	740±18	ns	C
	19.97	1080±28.2	b	A	767±53.6	b	B	1083±69	ns	A	924±86.9	ns	AB
Si	0	4600±814	ns	-	6766±1417	ns	-	5100±924	ns	-	5566±433	b	-
	5.44	8266±3218	ns	-	11966±2521	ns	-	4766±1507	ns	-	6700±737	ab	-
	19.97	8133±1827	ns	-	12700±1101	ns	-	7366±633	ns	-	8500±503	a	-
Ti	0	4±1.02	ns	-	5.±0.17	ns	-	4.16±0.14	ns	-	4.3±0.23	b	-
	5.44	4.8±0.63	ns	-	6.2±1.1	ns	-	5±0.93	ns	-	5.53±0.2	ab	-
	19.97	4.2±1.23	ns	-	7.3±0.2	ns	-	6.2±0.42	ns	-	6.6±0.56	a	-
K/Na	0	273±24.49	a	A	287±86.34	a	AB	153.7±39.7	a	AB	86.83±20.8	a	B
	5.44	177±96.1	a	A	121.8±29.71	a	A	97.3±8.06	a	A	7.43±1.99	b	B
	19.97	7.65±1.92	b	A	1.29±0.27	b	B	0.52±0.06	b	B	0.62±0.10	c	B
Ca/Na	0	239±6.34	a	ns	248±101.1	a	ns	293±48.57	a	ns	120±34.25	a	ns
	5.44	107±41.8	a	A	83.3±21.68	a	A	117±9.35	b	A	11.86±2.75	b	B
	19.97	3.57±0.91	b	A	0.72±0.15	b	B	0.46±0.056	c	B	0.43±0.06	c	B
Ca/K	0	0.86±0.08	ns	AB	0.77±0.16	ns	B	2.07±0.44	a	A	1.31±0.14	ab	AB

	5.44	0.72±0.15	ns	B	0.67±0.07	ns	B	1.17±0.08	ab	AB	1.63±0.17	a	A
	19.97	0.45±0.04	ns	B	0.54±0.06	ns	AB	0.86±0.02	b	A	0.72±0.16	b	AB
Si/K	0	0.73±0.1	ns	B	1.04±0.11	ns	AB	1.55±0.28	ns	A	1.59±0.09	a	A
	5.44	1.02±0.18	ns	ns	1.42±0.19	ns	ns	0.78±0.23	ns	ns	1.68±0.25	a	ns
	19.97	0.69±0.12	ns	B	1.12±0.06	ns	A	0.95±0.029	ns	AB	0.86±0.09	b	AB

Table S7. Tukey HSD (T_{HSD}) *post-hoc* test for the effects of treatment and harvest day on combustion indices base to acid ratio (Rb/a) and Base (%) of *M.* × *giganteus* leaves at 0, 5.44, 19.97 dS m^{-1} NaCl on harvest days 19, 32, 46 and 54. Different lowercase letters indicate significant differences between treatments (BT) for each harvest day; uppercase letters differences within treatment (WT) between harvest days at $p<0.05$; ns indicate no significant differences. Data are mean ± Standard Error (n=3).

Total element	Harvest days	19			32			46			54		
	NaCl	mean ± SE	T_{HSD} BT	T_{HSD} WT	mean ± SE	T_{HSD} BT	T_{HSD} WT	mean ± SE	T_{HSD} BT	T_{HSD} WT	mean ± SE	T_{HSD} BT	T_{HSD} WT
$R_{b/a}$	0	4.51±0.94	ns	ns	3.11±0.57	ns	ns	3.73±0.99	ns	ns	2.59±0.31	b	ns
	5.44	3.07±0.73	ns	ns	2.01±0.314	ns	ns	5.49±1.78	ns	ns	2.96±0.34	b	ns
	19.97	3.82±0.56	ns	B	3.49±0.43	ns	B	6.8±0.58	ns	A	6.52±0.079	a	A
Base (%)	0	4.15±0.16	b	A	4.17±0.07	b	AB	3.7±0.29	b	B	3.05±0.18	c	B
	5.44	4.45±0.32	b	ns	4.82±0.17	b	ns	4.55±0.13	b	ns	4.14±0.14	b	ns
	19.97	6.23±0.43	a	B	9.36±0.88	a	A	10.6±0.075	a	A	11.8±0.76	a	A
